# Rational Engineering of Non-Ubiquinone Containing *Corynebacterium glutamicum* for Enhanced Coenzyme Q_10_ Production

**DOI:** 10.3390/metabo12050428

**Published:** 2022-05-11

**Authors:** Arthur Burgardt, Ludovic Pelosi, Mahmoud Hajj Chehade, Volker F. Wendisch, Fabien Pierrel

**Affiliations:** 1 Genetics of Prokaryotes, Faculty of Biology and CeBiTec, Bielefeld University, 33615 Bielefeld, Germany; arthur.burgardt@uni-bielefeld.de; 2 University Grenoble Alpes, CNRS, UMR5525, VetAgro Sup, Grenoble INP, TIMC, 38000 Grenoble, France; ludovic.pelosi@univ-grenoble-alpes.fr (L.P.); mahmoud.hajj-chehade@univ-grenoble-alpes.fr (M.H.C.)

**Keywords:** coenzyme Q_10_ (CoQ_10_), ubiquinone, *Corynebacterium glutamicum*, metabolic engineering, Ubi complex, polyprenyl diphosphate synthase

## Abstract

Coenzyme Q_10_ (CoQ_10_) is a lipid-soluble compound with important physiological functions and is sought after in the food and cosmetic industries owing to its antioxidant properties. In our previous proof of concept, we engineered for CoQ_10_ biosynthesis the industrially relevant *Corynebacterium glutamicum*, which does not naturally synthesize any CoQ. Here, liquid chromatography–mass spectrometry (LC–MS) analysis identified two metabolic bottlenecks in the CoQ_10_ production, i.e., low conversion of the intermediate 10-prenylphenol (10P-Ph) to CoQ_10_ and the accumulation of isoprenologs with prenyl chain lengths of not only 10, but also 8 to 11 isopentenyl units. To overcome these limitations, the strain was engineered for expression of the Ubi complex accessory factors UbiJ and UbiK from *Escherichia coli* to increase flux towards CoQ_10_, and by replacement of the native polyprenyl diphosphate synthase IspB with a decaprenyl diphosphate synthase (DdsA) to select for prenyl chains with 10 isopentenyl units. The best strain UBI6-Rs showed a seven-fold increased CoQ_10_ content and eight-fold increased CoQ_10_ titer compared to the initial strain UBI4-Pd, while the abundance of CoQ_8_, CoQ_9_, and CoQ_11_ was significantly reduced. This study demonstrates the application of the recent insight into CoQ biosynthesis to improve metabolic engineering of a heterologous CoQ_10_ production strain.

## 1. Introduction

Coenzyme Q (CoQ), also referred to as ubiquinone, is a prenylated quinone compound that plays an essential role in the respiratory chain of eukaryotes and many prokaryotes. CoQ possesses certain chain lengths in different organisms defined by the number of isopentenyl units, e.g., CoQ_6_ in *Saccharomyces cerevisiae*, CoQ_8_ in *Escherichia coli*, and CoQ_10_ in humans. Next to its function in respiratory chains, CoQ serves as a lipid-soluble antioxidant that protects cellular membranes and lipoproteins from oxidative damage [1], as an activator of mitochondrial uncoupling proteins [2], and as a cofactor of several important enzymes such as mitochondrial dehydrogenases involved in different metabolic pathways [3]. Genetic CoQ_10_ deficiencies may cause various severe disorders, of which symptoms can sometimes be mitigated by CoQ_10_ supplementation [4,5,6]. Dietary supplementation has also shown beneficial effects in patients with cardiovascular and neurodegenerative diseases [7,8]. Especially in the food supplement [9] and cosmetic industries [10], CoQ_10_ has gained a large interest. Due to its challenging, low stereoselectivity yielding and expensive chemical synthesis, advanced semi-synthetic approaches have been developed [11]. However, microbial production offers a cheap and sustainable alternative owing to advances in the understanding of CoQ biosynthesis, metabolic engineering, biotechnological processes, and potent CoQ_10_ synthesizing bacteria, e.g., *Agrobacterium tumefaciens* and *Rhodobacter sphaeroides* [12,13,14].

**Figure 1:**
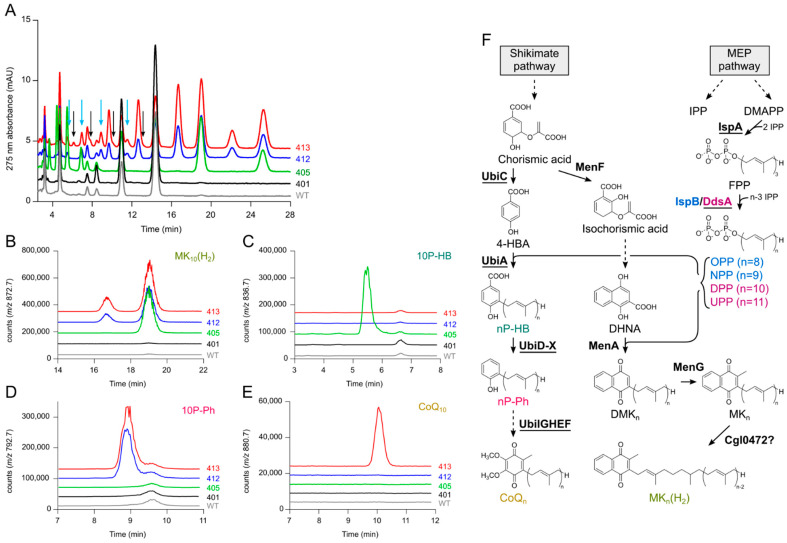
LC–MS analysis of quinone extracts of *C. glutamicum* strains WT, UBI401, UBI405, UBI412, and UBI413 to identify metabolic bottlenecks in CoQ_10_ production. (**A**) Overlay of UV chromatograms with black arrows pointing at CoQ_8-11_ peaks and blue arrows pointing at 8P-Ph–11P-Ph peaks to indicate the low flux from nP-Ph to CoQ_n_; (**B**–**E**) SIM overlays of NH_4_^+^ adduct of MK_10_(H_2_) (*m*/*z* = 872.7, **B**); NH_4_^+^ adduct of 10P-HB (*m*/*z* = 836.7, **C**); NH_4_^+^ adduct of 10P-Ph (*m*/*z* = 792.7, **D**); NH_4_^+^ adduct of CoQ_10_ (*m*/*z* = 880.7, **E**); (**F**) Metabolic pathway of CoQ_n_ and MK_n_(H_2_) biosynthesis. Enzymes are in bold, heterologous enzymes are underlined. As IspB mainly synthesizes NPP and OPP and DdsA mainly synthesizes DPP and UPP, the enzymes and corresponding direct products were marked with matching colors. The question mark indicates that the reaction attributed to Cgl0472 is not experimentally proven. MEP, methylerythritol phosphate; IPP, isopentenyl diphosphate; DMAPP, dimethylallyl diphosphate; FPP, farnesyl diphosphate; OPP, octaprenyl diphosphate; NPP, nonaprenyl diphosphate; DPP, decaprenyl diphosphate; UPP, undecaprenyl diphosphate; 4-HBA, 4-hydroxybenzoic acid; nP-HB, 3-n-prenyl-4-hydroxybenzoic acid; nP-Ph, 2-n-prenylphenol; CoQ_n_, coenzyme Q_n_/ubiquinone-n; DHNA, 1,4-dihydroxy-2-naphthoic acid; DMK_n_, demethylmenaquinone-n; MK_n_, menaquinone-n; MK_n_(H_2_), dihydromenaquinone-n; IspA, farnesyl diphosphate synthase; IspB, polyprenyl diphosphate synthase; DdsA, decaprenyl diphosphate synthase; UbiC, chorismate-pyruvate lyase; UbiA, 4-hydroxybenzoate octaprenyltransferase; UbiD-X, 3-octaprenyl-4-hydroxybenzoate decarboxylase and flavin prenyltransferase; UbiI-G-H-E-F, 2-octaprenylphenol hydroxylase, 2-octaprenyl-6-hydroxyphenol/2-octaprenyl-3-methyl-5-hydroxy-6-methoxy-1,4-benzoquinol methyltransferase, 2-octaprenyl-6-methoxyphenol hydroxylase, ubiquinone/menaquinone biosynthesis methyltransferase, 2-octaprenyl-3-methyl-6-methoxy-1,4-benzoquinol hydroxylase; MenF, isochorismate synthase; MenA, 1,4-dihydroxy-2-naphthoate octaprenyltransferase; MenG, demethylmenaquinone methyltransferase; Cgl0472, putative menaquinone oxidoreductase.

CoQ consists of a polysubstituted aromatic ring and a polyprenyl side chain. In bacteria, the aromatic precursor 4-hydroxybenzoate (4-HBA) is synthesized by cleavage of shikimate pathway-derived chorismate, while polyprenyl diphosphate originates from the methylerythritol phosphate (MEP) pathway. The number of isopentenyl diphosphate units added to farnesyl diphosphate by the polyprenyl diphosphate synthase defines the chain length of CoQ. The condensation of the polyprenyl side chain to 4-HBA is followed by multiple modifications of the aromatic ring in the late CoQ pathway to yield the final CoQ molecule (Figure 1F) [15]. Several metabolic engineering strategies have been applied to increase CoQ_10_ production in *R. sphaeroides*, including the upregulation of rate-limiting enzymes from the MEP and late CoQ pathways [16], increasing the NADH/NAD^+^ ratio and oxygen uptake [12], and decreasing the competing carotenoid synthesis [17]. The natural CoQ_8_ producer *E. coli* was engineered by the deletion of octaprenyl diphosphate synthase, encoded by *ispB*, and expression of a heterologous decaprenyl diphosphate synthase from *Paracoccus denitrificans*, encoded by *ddsA*, to produce a CoQ_10_ content of around 0.43 mg g^−1^ cell dry weight (CDW) under optimized cultivation conditions [18].

Recently, we metabolically engineered *Corynebacterium glutamicum*, a Gram-positive bacterium that solely possesses dihydromenaquinone (MK(H_2_)) and menaquinone (MK) [19], for the biosynthesis of CoQ [20]. This was achieved by deletion of the competing carotenoid pathway, establishment of 4-HBA and decaprenyl diphosphate (DPP) biosynthesis, and the heterologous expression of the *E. coli* CoQ pathway, encoded by *ubiADXIBGHEF*. This was the first instance of the transfer of CoQ biosynthesis to an organism that does not synthesize CoQ naturally, and it was especially important because *C. glutamicum* is a microbial host with high biotechnological relevance. Indeed, *C. glutamicum* is being used for the million-ton scale production of l-lysine and l-glutamate [21] and has been metabolically engineered to produce a variety of amino acids and amino acid-derived compounds such as l-2-hydroxyglutarate [22], l-DOPA [23], *N*-methylphenylalanine [24], and *N*-methylanthranilate [25]. Aromatic compounds such as protocatechuate [26] and 4-HBA [27,28] have been produced very efficiently, and *C. glutamicum* has also been employed for the production of isoprenoids such as patchoulol [29], astaxanthin [30], and α-carotene [31], which makes *C. glutamicum* a suitable host organism for the production of isoprenoid quinones such as CoQ_10_. In our recent study, however, limitations were observed in the CoQ_10_ synthesizing strain UBI413 as several unidentified supposable intermediates and side products, as well as the main product CoQ_10_, were formed. It was conceivable that UBI413 synthesized CoQ_8_ and CoQ_9_ due to endogenous polyprenyl diphosphate synthase activity, putatively encoded by *ispB* [32]. Moreover, 1,4-dihydroxy-2-naphthoate octaprenyltransferase MenA might accept DPP, resulting in the formation of MK_10_(H_2_) and MK_10_ in addition to native MK_9_(H_2_), MK_8_(H_2_), and MK_9_ [19].

In this study, we analyzed and identified the intermediates and side products in UBI413 and developed a strategy to reduce by-product formation and overcome bottlenecks in order to increase the flux towards CoQ_10_. Two alternative DPP synthases were compared against the DPP synthase from *P. denitrificans* that has been used previously, and the endogenous polyprenyl diphosphate synthase IspB was replaced with a DPP synthase to reduce the accumulation of 8-, 9- and 11-isoprenologs. The genes *ubiJ* and *ubiK*, encoding accessory factors for CoQ biosynthesis [33], were expressed to channel the flux by UbiI-G-H-E-F. In a combined approach, the CoQ_10_ content of the final strain was 7-fold increased, while the accumulation of intermediates and by-products was considerably reduced. Additionally, CoQ_10_ was produced using a hydrolysate from a wheat side stream as an alternative feedstock to demonstrate a sustainable production process.

## 2. Results

### 2.1. Identification of Accumulating Compounds in the Parent Strains

The chromatograms of the lipid extracts from strains UBI401, UBI405, UBI412, and UBI413 in our previous publication showed multiple peaks that remained unidentified [20] (Figure 1A). Using LC–MS, we tried to identify these compounds.

First, we suspected that the two main peaks observed in wild type (WT) and UBI401 corresponded to MK_8_(H_2_) and MK_9_(H_2_), which had previously been described in corynebacteria. The mass spectra of the compounds eluting at 11 and 14.4 min in WT cells showed ions corresponding to H^+^ and NH_4_^+^ adducts of MK_8_(H_2_) (*m*/*z* = 719.6 and 736.6, Figure S1A) and MK_9_(H_2_) (*m*/*z* = 787.6 and 804.6, Figure S1B). The UV spectra (not shown) were also characteristic of naphthoquinone species. Single ion monitoring (SIM) of the NH4^+^ adducts showed that MK_8_(H_2_) and MK_9_(H_2_) were indeed present in the lipid extracts of all strains, albeit in various amounts (Figure S1C,D).

Strain UBI405 expresses the decaprenyl synthase gene *ddsA* from *P. denitrificans* and the *ubiA* gene from *E. coli*, which encodes the polyprenyl transferase that prenylates 4-HBA. In comparison to UBI401, UBI405 showed several new peaks, two of them eluting late at 19 and 25.3 min and four others eluting early between 3.5 and 7 min (Figure 1A). The compounds eluting at 19 and 25.3 min displayed UV spectra characteristic of naphthoquinone species (not shown) and their mass spectra showed ions corresponding to H^+^ and NH_4_^+^ adducts of MK_10_(H_2_) (*m*/*z* = 855.7 and 872.7, Figure S2A) and MK_11_(H_2_) (*m*/*z* = 923.8 and 940.8, Figure S2B). SIM of the NH_4_^+^ adducts showed that MK_10_(H_2_) (Figure 1B) and MK_11_(H_2_) (Figure S2C) were absent in WT and strain UBI401 but present in the extracts of strains UBI405, UBI412, and UBI413, in agreement with the presence of *ddsA* in those later strains. These results show that expressing *ddsA* in *C. glutamicum* allows the synthesis of unnatural decaprenyl dihydromenaquinone as expected, but they also demonstrate poor specificity of DdsA from *P. denitrificans* since we also observed undecaprenyl dihydromenaquinone, MK_11_(H_2_) (Figure S2B,C). Together, our data show that strains UBI405, UBI412, and UBI413 synthesize four isoprenologs of MK(H_2_), ranging from MK_8_(H_2_) to MK_11_(H_2_) (Figures S1 and S2), with MK_10_(H_2_) and MK_11_(H_2_) being the most abundant in strains UBI412 and UBI413 (Figure S2D). Interestingly, the abundance of MK_8_(H_2_) and MK_9_(H_2_) increased in strain UBI401 compared to WT (Figure S2D), validating the engineering aimed at increasing FPP supply and flux in the shikimate pathway.

The compounds accumulated in strain UBI405 and eluting at 3.7, 4.5, 5.5, and 7 min showed ions compatible with NH_4_^+^ adducts of octaprenyl-4HBA (8P-HB, *m*/*z* = 700.5), nonaprenyl-4HBA (9P-HB, *m*/*z* = 768.6), decaprenyl-4HBA (10P-HB, *m*/*z* = 836.7), and undecaprenyl-4HBA (11P-HB *m*/*z* = 904.8) (Figure S3A–D). SIM revealed that these four compounds were detectable only in strain UBI405 (Figure 1C and Figure S3E–G), with isoprenologs 9 and 10 being the most abundant.

Strains UBI412 and UBI413 showed several new peaks compared to the other strains (Figure 1A) and LC–MS analysis identified two series of compounds: polyprenylphenols (nP-Ph) eluting between 5 and 12 min and menaquinones 8–11 (MK_8–11_) eluting between 9.5 and 23 min (Figures S4 and S5). We detected NH_4_^+^ adducts of polyprenylphenol composed of 9, 10, and 11 isoprene units at 7, 8.9, and 11.5 min, respectively (Figure S4A–C). The corresponding SIM showed the presence of these molecules only in strains UBI412 and UBI413 (Figure 1C and Figure S4D,E), consistent with the expression of UbiD and UbiX, allowing for decarboxylation of nP-HB from strain UBI405 into nP-Ph. We could not obtain an unambiguous detection of octaprenylphenol (8P-Ph, *m*/*z* = 656.5) because a co-eluting compound at 5.5 min exhibited a prominent signal at *m*/*z* = 654.6 (data not shown).

The compounds eluting at 9.7, 12.7, 16.5, and 22.2 min in strains UBI412 and UBI413 corresponded to fully unsaturated menaquinones 8–11 with mass spectra displaying characteristic H^+^ and NH_4_^+^ adducts (Figure S5).

Finally, strain UBI413 that expresses all the enzymes of the CoQ pathway was shown to produce CoQ_10_ (in agreement with our previous results [20], Figure 1E) and also CoQ_8_, CoQ_9_, and CoQ_11_ (Figure S6). It is worth noting that the peaks corresponding to CoQ_8-11_ were barely detectable in the 275 nm absorbance chromatogram (black arrows on Figure 1A), whereas those corresponding to nP-Ph were more prominent (blue arrows on Figure 1A). Since the molar absorption coefficient of 8P-Ph is about five-fold lower than that of CoQ_8_ [34], 8-11P-Ph are certainly more abundant than the corresponding CoQ_8–11_ in strain UBI413, denoting that the late steps of the CoQ pathway do not function optimally in strain UBI413.

In conclusion, we have now assigned all the peaks displayed in the 275 nm chromatograms of the lipid extracts of the strains previously published. These results suggested to us several options to increase CoQ_10_ biosynthesis in *C. glutamicum*, namely, (i) favor the accumulation of decaprenyl compounds over those with chains composed of 8, 9, or 11 prenyl units (ia—deletion of endogenous *ispB*, ib—screen for *ddsA* with higher specificity) and (ii) increase the overall efficiency of the CoQ pathway by expression of “accessory proteins”.

### 2.2. Deletion of ispB Diminishes Formation of 8- and 9-Isoprenologs

Due to native polyprenyl diphosphate synthase activity in *C. glutamicum*, presumably encoded by *ispB* [32], isoprenologs with a chain length of eight and nine prenyl units appeared in all quinone extracts in addition to the desired 10-isoprenologs (Figure 1F). When deletion of *ispB* was previously attempted in *C. glutamicum*, it remained unsuccessful as it is likely essential for menaquinone biosynthesis [32]. Here, we performed deletion of *ispB* with simultaneous replacement by *ddsA* from *P. denitrificans* in the strain UBI4, which was successful, yielding the viable strain UBI5. This strain and its isogenic parent strain were grown by microcultivation and examined for quinone content.

**Figure 2:**
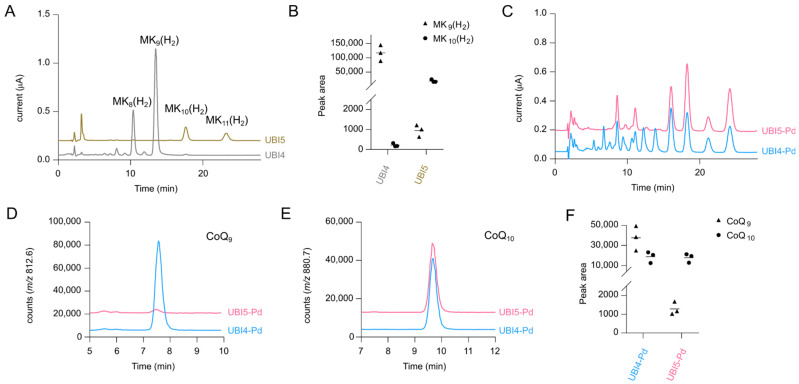
(**A**) Overlay of electrochemical detection (ECD) chromatograms from extracts of strains UBI4 and UBI5. The peaks corresponding to MK_8-11_(H_2_) are marked. (**B**) Quantification of MK_9_(H_2_) and MK_10_(H_2_) (MS peak area) in three independent samples of UBI4 and UBI5 cells. (**C**) Overlay of ECD chromatograms from extracts of strains UBI4-Pd and UBI5-Pd. (**D**,**E**) Overlay of SIM chromatograms for CoQ_9_ (NH_4_^+^ adduct *m*/*z* = 812.6, **D**), CoQ_10_ (NH_4_^+^ adduct *m*/*z* = 880.7, **E**). Chromatograms are representative of three independent samples. (**F**) Quantification of CoQ_9_ and CoQ_10_ (MS peak area) in three independent samples of UBI4-Pd and UBI5-Pd cells.

As the ECD chromatograms of UBI4 and UBI5 quinone extracts revealed, the latter strain synthesized no MK_8_(H_2_) and MK_9_(H_2_) at all and instead, to a much lesser extent, MK_10_(H_2_) and MK_11_(H_2_) were detected (Figure 2A,B), which serves as an indirect proof that IspB catalyzes octa- and nonaprenyl diphosphate formation in *C. glutamicum*. The MK_11_(H_2_) accumulation was a result of DdsA from *P. denitrificans* being unspecific regarding polyprenyl diphosphate synthesis activity, as observed previously in strain UBI413 (Figure S2C). Since menaquinone has vital functions in the cell, the influence of these drastic changes on growth behavior was monitored. Surprisingly, no significant difference in growth between both strains was observed in glucose minimal medium as the growth curves almost resembled the growth curve of the WT (Figure S7). Hence, in the next step, both strains were transformed with the plasmids necessary for CoQ_10_ production, pRG_Duet2-*ddsA_Pd_-ubiA*, pEC-XT99A-*ubiDIBX*, and pEKEx3-*ubiGHEF*, resulting in the strains UBI4-Pd (=UBI413) and UBI5-Pd, followed by cultivation in shake flasks and quinone extract analysis.

As can be seen from the ECD chromatograms from extracts of UBI4-Pd and UBI5-Pd, fewer peaks appeared for UBI5-Pd (Figure 2C). SIM chromatograms of NH_4_^+^ adducts display that the missing peaks comprised, i.a., CoQ_9_ (*m*/*z* = 812.6, Figure 2D), CoQ_8_ (*m*/*z* = 744.5, Figure S8A), and MK_9_(H_2_) (*m*/*z* = 804.6, Figure S8C) as expected. However, levels of CoQ_10_ (*m*/*z* = 880.7, Figure 2E,F), CoQ_11_ (*m*/*z* = 948.8, Figure S8B), and MK_10_(H_2_) (*m*/*z* = 872.7, Figure S8D) remained unchanged between the strains. Overall, the replacement of *ispB* with *ddsA* abolished the synthesis of unwanted 8- and 9-isoprenologs without any impact on cells’ growth, but it did not increase CoQ_10_ production. 

### 2.3. Screening of Different Decaprenyl Diphosphate Synthases

The expression of *ddsA* from *P. denitrificans* led to the formation of undesired 11-isoprenologs; thus, two other *ddsA* genes from *A. tumefaciens* and *R. sphaeroides* were screened for their by-product formation as these bacteria are among the best and most relevant CoQ_10_ production hosts [35,36]. The three genes were expressed in separate strains from the plasmids pRG_Duet2-*ddsA_Pd_-ubiA*, pRG_Duet2-*ddsA_At_-ubiA*, and pRG_Duet2-*ddsA_Rs_-ubiA*, together with those coding for the late CoQ pathway proteins, resulting in the strains UBI4-Pd, UBI4-At, and UBI4-Rs. The strains were cultivated in shake flasks and analyzed for their quinone content.

To estimate the differences in the formation of isoprenologs with different side chain lengths, ratios of peak areas from mass spectrometry analysis were calculated for several compounds (Table 1). The CoQ_10_/CoQ_11_ ratio was close to 1 in UBI4-Pd, revealing that DdsA from *P. denitrificans* is rather unspecific regarding polyprenyl diphosphate synthesis activity. On the other hand, extracts of UBI4-At contained almost no CoQ_11_ at all, resulting in a ratio of 145.4 ± 12.4. UBI4-Rs had a comparatively lower CoQ_10_/CoQ_11_ ratio of 7.6 ± 0.05, but a higher CoQ_10_/CoQ_9_ ratio than the other strains, which indicates that DdsA from *R. sphaeroides* synthesizes less nonaprenyl diphosphate as a side product than the other DdsA enzymes. This also reflected in the ratio of the intermediate prenylphenols 10P-Ph/9P-Ph (Table 1). The production of CoQ_10_ was not significantly improved, which might be due to a metabolic bottleneck downstream of the DdsA reaction. The results demonstrate that although all three DdsAs perform the same reaction, they differ in their precision to elongate FPP by a fixed number of seven IPP units. By this metric, DdsA enzymes of *A. tumefaciens* and *R. sphaeroides* are superior to that of *P. denitrificans*.

### 2.4. Expression of ubiJK Alleviates a Major Bottleneck

As recently shown, the *E. coli* enzymes UbiI-G-H-E-F, that catalyze the steps from prenylphenol to CoQ, form a soluble multiprotein complex with the accessory factors UbiJ and UbiK [33,37]. To find out if this complex might form in a heterologous environment, *C. glutamicum* in this case, and improve the flux in the late CoQ pathway, the genes *ubiJ* and *ubiK* from *E. coli* were integrated into the genome of UBI4 under control of the strong promoter of *actA* [38], yielding strain UBI4JK. Equipped with the necessary plasmids for CoQ_10_ biosynthesis (pRG_Duet2-*ddsA_Pd_-ubiA*, pEC-XT99A-*ubiDIBX*, and pEKEx3-*ubiGHEF*), quinones of the strains UBI4-Pd and UBI4JK-Pd were extracted after shake flask cultivation and subjected to LC–MS analysis as described above.

**Figure 3:**
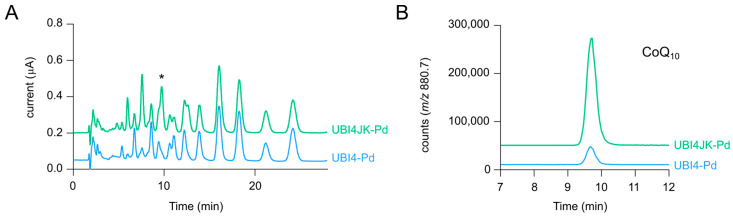
(**A**) Overlay of ECD chromatograms from extracts of strains UBI4-Pd and UBI4JK-Pd. * indicates the peak corresponding to CoQ_10_. (**B**) Overlay of SIM chromatograms for CoQ_10_ (NH_4_^+^ adduct *m*/*z* = 880.7). Chromatograms are representative of three independent samples.

Regarding growth of the strains UBI4 and UBI4JK without plasmids, no comparative experiment was performed; hence, the direct influence of *ubiJK* expression on growth was not evaluated. For strains UBI4-Pd and UBI4JK-Pd, however, no significant difference in growth rate or final biomass formation was observed (data not shown). The CoQ_10_/10P-Ph ratio, although not reflecting the stoichiometry of the two molecules, can serve as an indicator for the flux efficiency between the early pathway intermediate 10P-Ph and the final product CoQ_10_ (Table 2). While in UBI4-Pd the ratio was 0.3 ± 0.1, indicating a rather low flux, expression of *ubiJK* in UBI4JK-Pd increased the ratio in favor of CoQ_10_ production (1.5 ± 0.2). Consequently, the biomass yield, titer, and volumetric productivity increased around four-fold (Table 2). The improved CoQ_10_ production was also well visible in the ECD chromatograms where the peak corresponding to CoQ_10_ increased in the extract of UBI4JK-Pd (Figure 3A). The SIM chromatograms for the NH_4_^+^ adduct of CoQ_10_ underline the difference more clearly (Figure 3B). The increased flux in the late CoQ pathway also reflected in increased CoQ_8_ and CoQ_9_ levels (Figure S9A,B), while MK_9_(H_2_) and MK_10_(H_2_) levels remained almost unchanged (Figure S9C,D). In line with results showing that *E. coli ubiJ* and *ubiK* mutants contain no or a reduced amount of CoQ_8_ [33,39], our data demonstrate that the expression of *ubiJK* from *E. coli* is also important for efficient CoQ production in a heterologous host such as *C. glutamicum*.

### 2.5. Combinatorial Approach for Maximized CoQ_10_ Production

To study the combined effect of *ispB* replacement and *ubiJK* expression, the Δ*ispB*::P*_tuf_*-*ddsA* replacement was performed in UBI4JK, resulting in the strain UBI6. It was transformed with the plasmids pRG_Duet2-*ddsA_Pd_-ubiA*, pEC-XT99A-*ubiDIBX*, and pEKEx3-*ubiGHEF*, resulting in strain UBI6-Pd, followed by shake flask cultivation and LC–MS analysis of extracts. Compared to UBI4JK-Pd, the additional Δ*ispB*::P*_tuf_*-*ddsA* replacement had no significant effect on CoQ_10_ content, titer or volumetric productivity (Table 2). However, as observed for UBI5-Pd, CoQ_8_, CoQ_9_, and MK_9_(H_2_) amounts decreased severely (data not shown), which reflected in a CoQ_10_/CoQ_9_ ratio of 38.6 ± 1.9, even higher than for UBI5-Pd (14.4 ± 5.5). The deletion of endogenous *ispB* and the expression of *ubiJK* significantly improved the CoQ_10_ production and reduced the accumulation of side products in UBI6-Pd when compared to the initial strain UBI4-Pd.

**Figure 4:**
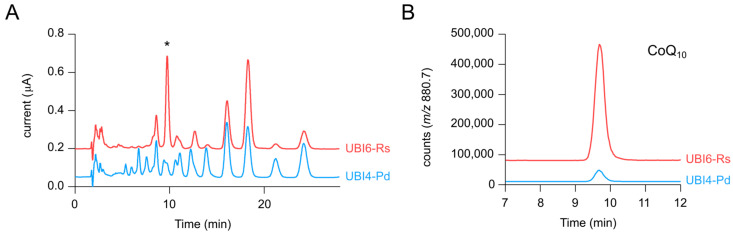
(**A**) Overlay of ECD chromatograms from extracts of strains UBI4-Pd and UBI6-Rs. * indicates the peak corresponding to CoQ_10_. (**B**) Overlay of SIM chromatograms for CoQ_10_ (NH_4_^+^ adduct *m*/*z* = 880.7). Chromatograms are representative of three independent samples.

We have shown that DdsA from *A. tumefaciens* and *R. sphaeroides* are more specific than DdsA from *P. denitrificans* towards the formation of 10P-HB compared to 9P-HB and 11P-HB (Table 1). In a final combinatorial approach, strains UBI6-At and UBI6-Rs were constructed for additive benefits and compared to UBI6-Pd. As observed previously, the strains with *ddsA* from *A. tumefaciens* and *R. sphaeroides* had an improved CoQ_10_/CoQ_11_ ratio of around 3.5 (Table 3). However, the ratio was not as high as for the strains UBI4-At and UBI4-Rs (Table 1), likely because of the chromosomal expression of the additional *ddsA* from *P. denitrificans* inserted in the Δ*ispB* locus. Regarding the CoQ_10_/CoQ_9_ ratio, UBI6-Pd and UBI6-Rs surprisingly shared a high ratio of around 40 compared to only 5.1 ± 0.4 for UBI6-At (Table 3). This indicates that the chromosomal expression of *ddsA* from *P. denitrificans* had less influence on CoQ_9_ production than on CoQ_11_ production. CoQ_10_ content, titer, and volumetric productivity were all twice as high in UBI6-Rs as for the other strains with values of 126.9 ± 10.7 µg g^−1^ CDW, 1.21 ± 0.12 mg L^−1^, and 16.8 ± 1.7 µg L^−1^ h^−1^, respectively. The CoQ_10_/10P-Ph ratio of UBI6-Rs was 60% higher than for the other two strains. The relative peak areas for 10P-Ph (Figure S10) were in a similar range for all of them despite the higher CoQ_10_ content of UBI6-Rs, indicating a pull effect for the intermediate 10P-Ph. This would be favorable if the flux from 10P-Ph to CoQ_10_ was further optimized in future strain engineering. To visualize the difference caused by the combinatorial approach, the ECD chromatograms and SIM chromatograms for the NH_4_^+^ adduct of CoQ_10_ are displayed in Figure 4. Compared to the initial strain UBI4-Pd, UBI6-Rs extracts contain much fewer and/or lower peaks, while the peak for CoQ_10_ has become the most prominent one. Nevertheless, the ECD chromatograms also show that MK_10_ and MK_10_(H_2_), eluting at 16.5 and 19 min, accumulated to considerable amounts as well. While the CoQ_10_ productivity reached here is still not competitive with productivity in native hosts such as *R. sphaeroides* [36], we provided a rational metabolic engineering approach in a non-native host, resulting in significantly higher CoQ_10_ production and lower by-product formation.

### 2.6. Influence of Growth Phase and Medium on CoQ_10_ Production

**Figure 5:**
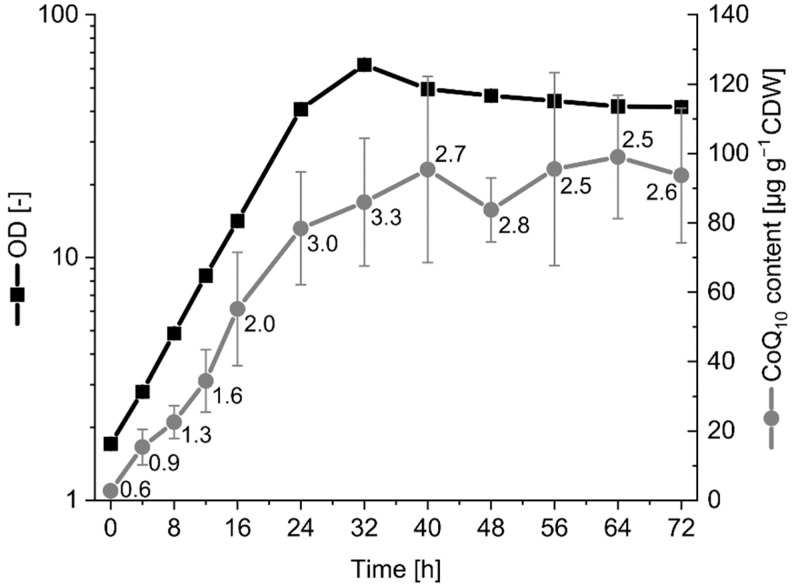
Growth and CoQ_10_ content of UBI6-Rs from shake flask cultivation. The cultivation was performed independently from the cultivation represented in Table 3. The numbers next to the grey data points indicate the CoQ_10_/CoQ_11_ ratios of relative peak areas from mass spectrometry analysis. Values and error bars represent means and standard deviations of 3 independent cultivations.

The best strain, UBI6-Rs, was cultivated for a time-resolved analysis of CoQ_10_ production (Figure 5). The cells grew with a specific growth rate of 0.13 h^−1^ in the first 24 h and the biomass reached its peak at 32 h with an optical density at 600 nm (OD_600_) of 62 (15.5 g L^−1^ CDW). The kinetics of growth and CoQ_10_ production largely overlapped with the CoQ_10_ content reaching 95 µg g^−1^ CDW after 40 h and remaining around that level until the end of cultivation. Notably, the CoQ_10_/CoQ_11_ ratio was rather low at the beginning with 0.6 but increased steadily during exponential growth to a maximum of 3.3 at 32 h (Figure 5). The strain UBI6-Rs carries the *ddsA* gene from *P. denitrificans* that is expressed constitutively in the *ispB* locus, and the *ddsA* gene from *R. sphaeroides*, expressed from the vector pRG_Duet2 upon induction by isopropyl-β-d-1-thiogalactopyranoside (IPTG) at the beginning of the cultivation. As shown before, expression of *ddsA* from *R. sphaeroides* promotes higher CoQ_10_ production with lower accumulation of CoQ_11_ than *ddsA* from *P. denitrificans*, which explains the low CoQ_10_/CoQ_11_ ratio at the beginning and its increase over time.

After CoQ_10_ production was successfully improved, the strain UBI6-Rs was cultivated with a hydrolysate of the alternative feedstock wheat side stream concentrate (WSCH) [40]. In a sustainable circular economy, side streams of industrial production processes can provide excellent alternative feedstocks for microbial production containing macro- and micronutrients [41]. Here, WSCH was supplemented with the nitrogen source ammonium sulfate and the buffer 3-(*N*-morpholino) propanesulfonic acid (MOPS) for cultivation of UBI6-Rs in a microcultivation system, CGXII minimal medium was used for comparison. LC–MS analysis confirmed that the CoQ_10_ concentration in WSCH medium before cultivation was below the detection limit of 1 nM. In the microcultivation system used for this comparison, the CoQ_10_ content, titer, and volumetric productivity in CGXII minimal medium were lower than in shake flasks (Table 3). The CoQ_10_ content obtained in microcultivation with WSCH medium was about 40% of that in microcultivation with CGXII medium (Table 3). As microcultivation with WSCH medium supported growth to a higher biomass concentration, the titer and volumetric productivity were about 55% of the values obtained in microcultivation with CGXII medium (Table 3). Moreover, by-product and intermediate formation in WSCH were mostly lower compared to CGXII medium as indicated by the CoQ_10_/CoQ_11_ and CoQ_10_/10P-Ph ratios (Table 3).

To conclude, although *C. glutamicum* is not a natural producer of any ubiquinone, we were able to identify metabolic bottlenecks in the initial metabolically engineered producer UBI4-Pd and to optimize the strain for seven-fold increased CoQ_10_ content and eight-fold increased CoQ_10_ titer with considerably lower by-product formation.

## 3. Discussion

The biosynthesis of CoQ is complex and has not been fully elucidated after 60 years of research, making its transfer to an organism without CoQ biosynthesis challenging. Previously, we set the foundation for heterologous CoQ_10_ production in *C. glutamicum* by expression of genes, fulfilling the minimum requirements for CoQ biosynthesis [20]. In this study, our goal was to identify metabolic bottlenecks by LC–MS analysis and to alleviate them by genetic engineering. The strategy comprised increasing flux by expression of accessory factor genes *ubiJ* and *ubiK*, deleting the native polyprenyl diphosphate synthase IspB, and expressing the best decaprenyl diphosphate synthase to concentrate isoprenologs production towards CoQ_10_.

In *E. coli*, UbiJ and UbiK induce the formation of a Ubi complex with UbiI-G-H-E-F that catalyzes the reactions of the late CoQ pathway [37]. Here, expression of *ubiJK* increased CoQ_10_ production four-fold. It is likely that, although not proven biochemically, this may have led to the formation of a Ubi complex in the heterologous host *C. glutamicum*. While CoQ_10_ production was substantially improved, 10P-Ph was still abundant in the strain UBI6-Rs, indicating that flux through the putative Ubi complex did not reach its full potential. One reason might be that the *E. coli* Ubi proteins we expressed in *C. glutamicum* are not well suited to modifying CoQ intermediates with a decaprenyl side chain because they naturally operate on compounds with an octaprenyl chain. Therefore, expressing Ubi proteins from a bacterium that naturally produces CoQ_10_ could improve the flux between 10P-Ph and CoQ_10_. Another reason might lie in the subunit stoichiometry of the Ubi complex. It has been shown in *E. coli* that synthesis rates of proteins that belong to a multiprotein complex are proportional to the subunit stoichiometry of their corresponding complexes [42] in order to save cellular resources and to avoid protein aggregation and misfolding [43]. Not only do the synthesis rates of the Ubi complex-associated proteins UbiK-J-I-G-H-E-F differ from each other [42], which is indicative of heterogeneous stoichiometry, but also UbiK and UbiJ were found to associate in a heterotrimeric UbiK_2_–UbiJ_1_ complex [33] and several Ubi proteins interacted with themselves [37]. This is in contrast to our cloning strategy in which *ubi* genes were expressed in artificial polycistronic operons and lacked any regulation, leading to disproportional protein abundances in relation to the Ubi complex stoichiometry. Expression fine-tuning would be a way to change expression levels of the single components of the Ubi complex, e.g., by the use of a promoter library [44], adjustment of transcriptional initiation rates using artificial ribosome binding sites [45], introduction of multiple gene copies into the genome [31,46], or changing the order of genes in the polycistronic operons [47]. In the natural CoQ_10_ producer *R. sphaeroides*, metabolic bottlenecks were identified to be UbiE, UbiH, and UbiG. Three different bottleneck elimination strategies were tested, among which fusion of UbiE and UbiG and localization of the fused protein onto the membrane via *pufX* linker gave the best results with a titer of 108.5 mg L^−1^ after 96 h of cultivation, a CoQ_10_ content of 8.9 mg g^−1^ CDW, and a volumetric productivity of 1.13 mg L^−1^ h^−1^ [16]. However, it is questionable if this strategy would be effective in our case due to differences in the molecular mechanism of CoQ biosynthesis between *E. coli* and *R. sphaeroides* enzymes. A multienzyme complex has not been confirmed for *R. sphaeroides* CoQ biosynthesis, and proteins homologous to *E. coli* UbiI, UbiG, UbiH, UbiE, and UbiF exist in *R. sphaeroides*, but none to the accessory factors UbiJ and UbiK according to Protein BLAST analysis. It should be mentioned that overexpression of *ubiJ* and *ubiK* in *E. coli* led to decreased CoQ_8_ content and four-fold and two-fold increases in 8P-Ph and 2-decaprenyl-3-methyl-6-methoxy-1,4-benzoquinol levels, respectively, presumably as a consequence of sequestration of these CoQ_8_ intermediates [37]. Thus, expression strength of *ubiJ* and *ubiK* seems to influence the flux from 10P-Ph to CoQ_10_ quite dramatically and should be adjusted accordingly.

Moreover, an increase in MK_10_ and MK_10_(H_2_) amounts was observed with expression of *ddsA* from *R. sphaeroides* in both background strains UBI4 and UBI6. We propose that Cgl0472 is a menaquinone oxidoreductase that reduces a double bond in the chain of MK, resulting in MK(H_2_) (Figure 1F), because it shares 51% sequence identity with menaquinone oxidoreductase MenJ from *Mycobacterium tuberculosis* [48]. The accumulation of fully unsaturated menaquinones might be caused by the inhibition of Cgl0472; however, this requires further investigation. Irrespective of the fact that the growth of *C. glutamicum* was not influenced by the different chain length of menaquinone, MK_10_ and MK_10_(H_2_) are competing by-products to CoQ_10_ and should be limited in favor of increased CoQ_10_ production. In *E. coli*, the competitive MK biosynthesis was blocked by the deletion of 1,4-dihydroxy-2-naphthoate (DHNA) octaprenyltransferase gene *menA*, ensuring that octaprenyl diphosphate would only be used to prenylate the CoQ intermediate 4-HBA, which led to increased CoQ_8_ content by 81% [49] and squalene content by 18% [50]. In *C. glutamicum*, however, MK is the only natural isoprenoid quinone and therefore vital. The downregulation of *menA* in *C. glutamicum* might allow flux into MK biosynthesis to be lowered without impacting growth. Analogously, in *R. sphaeroides*, the competitive carotenoid biosynthesis was downregulated, resulting in 28% increased CoQ_10_ production, since the complete disruption of carotenogenesis impaired both growth and CoQ_10_ production [17]. Nevertheless, it could be challenging to determine the right balance to lower the flux into MK biosynthesis as much as possible while maintaining growth and, thus, CoQ_10_ productivity. Alternatively, specific MenA inhibitors can be used to reduce MenA activity. Several drugs, among them an allylaminomethanone class of compounds, have been identified to inhibit MenA of *Mycobacterium tuberculosis*, acting as demethylmenaquinone (DMK) mimics [51,52]. The benefit of this approach is that different inhibitors can be tested and the optimal dose can be found comparatively fast, rendering the need for *menA* expression fine-tuning superfluous, if the inhibitor is not costly and can be used at a larger scale as well. Furthermore, quorum sensing provides another alternative to plain downregulation of *menA*. Being able to maintain regular expression of *menA* in the early growth phase and reducing it with increasing cell density would prevent growth deficits caused by menaquinone deficiency and reduce menaquinone accumulation. Liu et al. adapted the ComQXPA-P*_srfA_* quorum sensing system of *Bacillus subtilis* to *C. glutamicum* such that P*_srfA_*-controlled transcription of an *hfq*-sRNA complementary to a target gene was activated with high cell density, leading to silencing of the target gene by its complementary sRNA. In addition, a library of synthetic P*_srfA_* promoters was established to modulate the expression of the *hfq*-sRNA [53], allowing for optimized control over *menA* expression.

In order to improve precursor supply for CoQ_10_ production, overexpression of the MEP pathway genes *dxs* and *idi* is a common way to increase flux towards IPP and DMAPP and has been shown to increase patchoulol production in an engineered *C. glutamicum* strain [29]. Other strategies aim at the supply and distribution of the molecules of the MEP pathway entry point, glyceraldehyde 3-phosphate and pyruvate [54], e.g., by the modification of central carbon metabolism [55], CRISPRi-mediated repression [56], and increase in the NAD(P)H pool [57]. A different kind of approach is membrane engineering that involves the expression of proteins with membrane-bending properties and the overall increase in membrane synthesis to expand the membrane surface area and storage capacity for CoQ_10_. In *E. coli*, the monoglucosyldiacylglycerol synthase Almgs was overexpressed to induce the formation of membrane stacks or tubules and intracellular membrane vesicles, and the genes *plsB* and *plsC* were overexpressed to increase glycerophospholipid biosynthesis, which synergistically increased β-carotene production 2.9-fold [58]. However, media composition and cultivation conditions are potent factors as well and should be considered to improve productivity. For CoQ_10_ production, strategies such as controlling a low sucrose concentration during fed-batch fermentation of *A. tumefaciens* [59] or the cultivation of *R. sphaeroides* under phosphate limitation [60] proved to be very effective. In this study, the standard minimal medium CGXII for *C. glutamicum* [19] was used. Since it was designed for the production of amino acids, it contains a high concentration of nitrogen that should be tuned down in case of production of the nitrogen-free CoQ_10_. The reduction of nitrogen to 10% and of glucose to 50% increased the production of *N*-methylphenylalanine by *C. glutamicum* and reduced by-product formation [24]. As there are numerous CGXII components, and macro and trace elements, statistical methods such as response surface methodology help to find optimized conditions by using the proper design of experiments as was demonstrated for glutamate production in *C. glutamicum* [61]. Media optimization can also be employed to generate high cell densities in cultures, which is especially interesting for cell-bound products such as CoQ_10_. In a recent study, lignocellulose-derived acetate was utilized as a sole carbon source and as acid pH titrant, while urea was fed as a nitrogen source. By dynamical adaptation of the C/N feeding ratio, a maximal cell dry weight of 80.2 g L^−1^ was achieved [62].

With respect to alternative feedstocks that are not competitive with food or feed, *C. glutamicum* has been employed and engineered for many different substrates. Here, we demonstrated the successful production of CoQ_10_ from a wheat side stream-based hydrolysate that has been utilized previously for the production of 5-aminovalerate [40] and l-2-hydroxyglutarate [22]. As well as the wheat side stream, access to numerous monomeric and polymeric carbon sources has been established, e.g., xylose, arabinose, mannose, starch, lignocellulose, *N*-acetylglucosamine, and alginate, which can be derived from hydrolysates of sustainable second generation feedstocks such as spent sulfite liquor, *Miscanthus* biomass, brown seaweed, corn straw, rice straw, and shrimp waste [63,64,65,66].

Taken together, we demonstrated how CoQ_10_ production can be established in the non-ubiquinone containing organism *C. glutamicum* and optimized substantially by applying current knowledge about CoQ biosynthesis to establish its efficient production. Although the achieved CoQ_10_ content is not yet competitive with natural producers such as *R. sphaeroides*, our strain holds the potential for further improvements with regards to metabolic engineering, media and cultivation conditions, and cell density. Its well-established genetic tools, systems metabolic engineering, and insights into sustainable production processes make *C. glutamicum* an attractive organism for the production of high value-added compounds such as CoQ_10_.

## 4. Materials and Methods

### 4.1. Bacterial Strains and Growth Conditions

All bacterial strains used in this study are listed in Table 4. *E. coli* DH5α [67] was used for plasmid construction, *E. coli* S17-1 [68] was used for transfer of suicide vectors by trans-conjugation prior to chromosomal gene replacements, *C. glutamicum* UBI4 [20] was used for strain construction. Pre-cultures of *E. coli* and *C. glutamicum* were inoculated from fresh LB or BHI agar plates and cultivated in lysogeny broth (LB) and brain heart infusion (BHI) medium at 37 °C and 30 °C in non-baffled and baffled shake flasks on a rotary shaker at 180 rpm and 120 rpm, respectively. When appropriate, kanamycin (25 µg mL^−1^), spectinomycin (100 µg mL^−1^), and tetracycline (5 µg mL^−1^) were added to the media and plates. For production experiments, *C. glutamicum* cells from pre-cultures were washed with TN buffer pH 6.3 (10 mM Tris-HCl, 150 mM NaCl) and inoculated to an OD_600_ of 1 in 50 mL CGXII minimal medium [19] in 500 mL shake flasks. When specified, cultivations were performed in a BioLector microcultivation system (m2p-labs, Baesweiler, Germany) in 3.2 mL FlowerPlates at 1100 rpm and 30 °C and with filling volumes of 1 mL. The minimal medium was supplemented with 40 g L^−1^ glucose as sole carbon source, 1 mM IPTG to induce gene expression of *ddsA* from pRG_Duet2 [69] and all genes from pEC-XT99A [70] and pEKEx3 [71], 0.25 µg mL^−1^ anhydrotetracycline (ATc) to induce gene expression of *ubiA* from pRG_Duet2, and respective antibiotics. For cultivation in WSCH medium, 80% (*v*/*v*) hydrolysate (from 190 g L^−1^ WSC, [40]) was supplemented with inducers and antibiotics as described above, 20 g L^−1^ ammonium sulfate and 42 g L^−1^ MOPS to a final glucose concentration of 33.2 g L^−1^. OD_600_ was measured using a V-1200 spectrophotometer (VWR, Radnor, PA, United States). After 72 h, cells were harvested and stored at −20 °C. 

### 4.2. Molecular Genetic Techniques and Strain Construction

Standard molecular genetic techniques were performed as described [72]. Competent *E. coli* cells were prepared with the RbCl method and transformed by heat shock [72]. Competent *C. glutamicum* cells were prepared in NCM medium, with the exception of dl-threonine, according to an optimized transformation protocol [73]. Cells were transformed using electroporation at 2.5 kV, 200 Ω, and 25 µF. PCR amplification was performed with Phusion High-Fidelity DNA polymerase according to the manufacturer (New England Biolabs, Hitchin, UK). All plasmids are listed in Table 5 and were constructed via Gibson Assembly [74], using DNA fragments created with the primers specified in Table 6.

The *ddsA* genes were amplified from genomic DNA from *A. tumefaciens* C58 and *R. sphaeroides* ATH 2.4.1 (DSM 158); pRG_Duet2 was restricted with *BamHI* for insertion of *ddsA* and with *NheI* for insertion of *ubiA* as described [20]. The pK19*mobsacB* plasmids were constructed in two steps by amplification of the flanking regions of *actA* and *ispB* from genomic DNA of *C. glutamicum* ATCC 13,032 and restriction of pK19*mobsacB* with *BamHI*. In the second step, the plasmids pK19*mobsacB*-Δ*actA* and pK19*mobsacB*-Δ*ispB* were restricted in the newly generated restriction sites *BamHI* and *BcuI*, respectively, between the flanking regions. The genes *ubiJ* and *ubiK* were amplified from genomic DNA from *E. coli* K-12 MG1655; P*_tuf_*-*ddsA_Pd_* was amplified from pSH1-*ddsA_Pd_*, which was constructed before by restriction of pSH1 [76] with BamHI and amplification of *ddsA* from genomic DNA of *P. denitrificans*. Correct sequences were confirmed by sequencing of inserts. Gene replacements were performed by using the suicide vector pK19*mobsacB* and two-step homologous recombination as described [77]. Transfer of the vectors by trans-conjugation using S17-1 as donor strain and selection of the mutants was conducted as described [19]. Successful replacements were verified by PCR and sequencing with the primers specified in Table 6.

### 4.3. Quinone Extraction and Analysis

Pellets of *C. glutamicum* cells (10–25 mg) were suspended in cold PBS buffer in Eppendorf tubes. Cells were centrifuged at 13,000 rpm for 2 min at 4 °C, the supernatant was eliminated, and the wet weight of the pellet was determined. Glass beads (100 μL), 50 μL of 0.15 M KCl, and a volume of 2 mM MK_7_ solution (used as an internal standard, Sigma-Aldrich) proportional to the wet weight (2 μL/mg) were added to cell pellet. Quinone extraction was performed by adding 0.6 mL of methanol, vortexing for 10 min, then adding 0.4 mL of petroleum ether (boiling range 40–60 °C) and vortexing for 3 min. The phases were separated by centrifugation at 1 min, 5000 rpm. The upper petroleum ether layer was transferred to a fresh tube. Petroleum ether (0.4 mL) was added to the glass beads and methanol-containing tube, and the extraction was repeated. The petroleum ether layers were combined and dried under nitrogen. The dried samples were stored at −20 °C and were resuspended in 100 μL ethanol. Aliquots corresponding to 2 mg of cells’ wet weight were analyzed by reversed-phase HPLC with a C18 column (Betabasic-18, 5 mm, 4.6 × 150 mm; Thermo Scientific) at a flow rate of 1 mL/min with a mobile phase composed of 25% isopropyl alcohol, 20% ethanol, 45% methanol, and 10% of a mix of 90% isopropyl alcohol/10% ammonium acetate (1 M)/0.1% formic acid. Hydroquinones present in samples were oxidized with a precolumn 5020 guard cell set in oxidizing mode (E, +650 mV). Quinones were monitored by in-line UV detection (247 and 275 nm), by electrochemical detection (ECD) with an ESA Coulochem III electrochemical detector equipped with a 5011A analytical cell (E1, −650 mV; E2, +650 mV), and by mass spectrometry (MS) with an MSQ Plus spectrometer. The flow was divided after the diode array detector with an adjustable split valve (Analytical Scientific Instruments) to allow simultaneous EC (60% of the flow) and MS (40% of the flow) detections. The MSQ Plus was used in positive mode (probe temperature 400 °C, cone voltage 80 V). MS spectra were recorded between *m*/*z* 550 and 1000 with a scan time of 0.3 s, and single ion monitoring (NH_4_^+^ adducts, scan time 0.2 s) detected the following compounds: 8P-Ph, *m*/*z* 656.1–657.1, 3–6 min; MK_7_, *m*/*z* 666.0–667.0, 5–9 min; 8P-HB, *m*/*z* 700.0–701.0, 2.5–6 min; 9P-Ph, *m*/*z* 724.1–725.1, 5–9 min; MK_8_, *m*/*z* 734.0–735.0, 7–12 min; MK_8_(H_2_), *m*/*z* 736.0–737.0, 8–13 min; CoQ_8_, *m*/*z* 744.0–745.0, 3–8 min; 9P-HB, *m*/*z* 768.1–769.1, 2.5–6 min; 10P-Ph, *m*/*z* 792.2–793.2, 7–11 min; MK_9_, *m*/*z* 802.1–803.1, 10–14 min; MK_9_(H_2_), *m*/*z* 804.1–805.1, 11–16 min; CoQ_9_, *m*/*z* 812.1–813.1, 5–10 min; 10P-HB, *m*/*z* 836.2–837.2, 3–8 min; 11P-Ph, *m*/*z* 860.3–861.3, 9–13 min; MK_10_, *m*/*z* 870.2–871.2, 13–20 min; MK_10_(H_2_), *m*/*z* 872.2–873.2, 14–21 min; CoQ_10_, *m*/*z* 880.2–881.2, 7–12 min; 11P-HB, *m*/*z* 904.3–905.3, 5–9 min; MK_11_, *m*/*z* 938.3–939.3, 17–23 min; MK_11_(H_2_), *m*/*z* 940.3–941.3, 20–27 min; CoQ_11_, *m*/*z* 948.3–949.3, 11–15 min. Calculation of CoQ_10_ content was based on 1 g cell wet weight being equivalent to 0.25 g cell dry weight (CDW) [78].

## Figures and Tables

**Table 1:** Ratios of relative peak areas from mass spectrometry analysis and CoQ_10_ biomass yields, titers, and volumetric productivities for the strains UBI4-Pd, UBI4-At, and UBI4-Rs.

Strain	10P-Ph/ 9P-Ph	CoQ_10_/ CoQ_9_	CoQ_10_/ CoQ_11_	Y_x_ (µg g^−1^ CDW)	Titer (mg L^−1^)	Vol. Productivity (µg L^−1^ h^−1^)
UBI4-Pd	1.1 ± 0.1	0.5 ± 0.0	1.2 ± 0.1	18.2 ± 5.4	0.15 ± 0.05	2.1 ± 0.6
UBI4-At	1.2 ± 0.3	0.6 ± 0.2	145.4 ± 12.4 ***	21.3 ± 4.6	0.14 ± 0.04	2.0 ± 0.6
UBI4-Rs	1.6 ± 0.2 **	0.9 ± 0.1 **	7.6 ± 0.0 ***	24.9 ± 5.9	0.18 ± 0.04	2.5 ± 0.6

Statistical significance of values compared with values of UBI4-Pd is based on a two-sided unpaired Student’s *t*-test (**: *p* ≤ 0.01; ***: *p* ≤ 0.001).

**Table 2:** Ratios of relative peak areas from mass spectrometry analysis and CoQ_10_ biomass yields, titers, and volumetric productivities for the strains UBI4-Pd, UBI4JK-Pd, UBI5-Pd, and UBI6-Pd.

Strain	CoQ_10_/ 10P-Ph	CoQ_10_/ CoQ_9_	Y_x_ (µg g^−1^ CDW)	Titer (mg L^−1^)	Vol. Productivity (µg L^−1^ h^−1^)
UBI4-Pd	0.3 ± 0.1	0.5 ± 0.0	18.2 ± 5.4	0.15 ± 0.05	2.1 ± 0.6
UBI4JK-Pd	1.5 ± 0.2 ***	0.7 ± 0.1 *	78.0 ± 12.0 **	0.64 ± 0.08 ***	9.0 ± 1.1 ***
UBI5-Pd	0.2 ± 0.1	14.4 ± 5.5 *	17.3 ± 4.4	0.15 ± 0.04	2.1 ± 0.6
UBI6-Pd	1.2 ± 0.2 **	38.6 ± 1.9 ***	69.6 ± 9.4 **	0.58 ± 0.06 ***	8.0 ± 0.9 ***

Statistical significance of values compared with values of UBI4-Pd is based on a two-sided unpaired Student’s *t*-test (*: *p* ≤ 0.05; **: *p* ≤ 0.01; ***: *p* ≤ 0.001).

**Table 3:** Ratios of relative peak areas from mass spectrometry analysis and CoQ_10_ biomass yields, titers, and volumetric productivities for the strains UBI6-Pd, UBI6-At, and UBI6-Rs. In addition, UBI6-Rs was cultivated in a BioLector microcultivation system in CGXII medium (same as before) and WSCH medium.

Strain/ medium	CoQ_10_/ 10P-Ph	CoQ_10_/ CoQ_9_	CoQ_10_/ CoQ_11_	Y_x_ (µg g^−1^ CDW)	Titer (mg L^−1^)	Vol. Productivity (µg L^−1^ h^−1^)
UBI6-Pd	1.2 ± 0.2	38.6 ± 1.9	1.5 ± 0.0	69.6 ± 9.4	0.58 ± 0.06	8.0 ± 0.9
UBI6-At	1.2 ± 0.2	5.1 ± 0.4 **	3.5 ± 0.5 **	64.3 ± 4.6	0.61 ± 0.04	8.4 ± 0.6
UBI6-Rs	1.9 ± 0.5	41.6 ± 3.4	3.4 ± 0.2 ***	126.9 ± 10.7 **	1.21 ± 0.12 **	16.8 ± 1.7 **
Microcultivation of UBI6-Rs in CGXII medium and WSCH medium						
CGXII	1.0 ± 0.2	55.4 ± 7.2	4.2 ± 0.1	92.2 ± 17.2	0.89 ± 0.15	12.3 ± 2.1
WSCH	1.5 ± 0.2	31.4 ± 1.1	8.8 ± 0.6	37.7 ± 7.4	0.49 ± 0.08	6.8 ± 1.2

Statistical significance of values compared with values of UBI6-Pd is based on a two-sided unpaired Student’s *t*-test (**: *p* ≤ 0.01; ***: *p* ≤ 0.001).

**Table 4:** Bacterial strains used in this study.

Strains	Description	Source
*Corynebacterium glutamicum*		
WT	*C. glutamicum* wild-type strain ATCC 13032	ATCC
UBI4	WT with following modifications: Δ*crtOP* (cg0717-cg0723), Δ*idsA* (cg2384), Δ*crtB2I’I2* (cg2668-cg2672), LP4::P*_tuf_*-*ispA* (*ispA* from *E. coli*), Δ*pobA* (cg1226), Δ*pcaHG*::P*_sod_*-*ubiC*^FBR^ (cg2631-cg2630, *ubiC*^L31A^ *from E. coli*), Δ*vdh*::P*_ilvC_*-*aroG*^FBR^ (cg2953, *aroG*^D146N^ from *E. coli*), Δ*qsuABCD*::P*_tuf_*-*qsuC* (cg0501-cg0504); named UBI400 in [20]	[20]
UBI401	UBI4 carrying pRG_Duet2, pEC-XT99A, and pEKEx3	This work
UBI405	UBI4 carrying pRG_Duet2-*ddsA_Pd_-ubiA*, pEC-XT99A, and pEKEx3	This work
UBI412	UBI4 carrying pRG_Duet2-*ddsA_Pd_-ubiA*, pEC-XT99A-*ubiDIBX*, and pEKEx3	This work
UBI4-Pd	UBI4 carrying pRG_Duet2-*ddsA_Pd_-ubiA*, pEC-XT99A-*ubiDIBX*, and pEKEx3-*ubiGHEF*; named UBI413 in [20]	[20]
UBI4-At	UBI4 carrying pRG_Duet2-*ddsA_At_-ubiA*, pEC-XT99A-*ubiDIBX*, and pEKEx3-*ubiGHEF*	This work
UBI4-Rs	UBI4 carrying pRG_Duet2-*ddsA_Rs_-ubiA*, pEC-XT99A-*ubiDIBX*, and pEKEx3-*ubiGHEF*	This work
UBI5	Δ*ispB*::P*_tuf_*-*ddsA_Pd_* mutant of UBI4	This work
UBI5-Pd	UBI5 carrying pRG_Duet2-*ddsA_Pd_-ubiA*, pEC-XT99A-*ubiDIBX*, and pEKEx3-*ubiGHEF*	This work
UBI4JK	Δ*actA*::*ubiJK* mutant of UBI4	This work
UBI4JK-Pd	UBI4JK carrying pRG_Duet2-*ddsA_Pd_-ubiA*, pEC-XT99A-*ubiDIBX*, and pEKEx3-*ubiGHEF*	This work
UBI6	Δ*ispB*::P*_tuf_*-*ddsA_Pd_* mutant of UBI4JK	This work
UBI6-Pd	UBI6 carrying pRG_Duet2-*ddsA_Pd_-ubiA*, pEC-XT99A-*ubiDIBX*, and pEKEx3-*ubiGHEF*	This work
UBI6-At	UBI6 carrying pRG_Duet2-*ddsA_At_-ubiA*, pEC-XT99A-*ubiDIBX*, and pEKEx3-*ubiGHEF*	This work
UBI6-Rs	UBI6 carrying pRG_Duet2-*ddsA_Rs_-ubiA*, pEC-XT99A-*ubiDIBX*, and pEKEx3-*ubiGHEF*	This work
*Escherichia coli*		
DH5α	*F-thi-1 endA1 hsdr17*(*r-*, *m-*) *supE44 1lacU169* (Φ*80lacZ1M15*) *recA1 gyrA96*	[67]
S17-1	*recA pro hsdR* RP4-2-Tc::Mu-Km::Tn7	[68]

**Table 5:** Plasmids used in this study.

Plasmids	Description	Source
pRG_Duet2	Kan^R^, P*_tac_*, *lacI^q^*, P*_tetR_*_/*tetA*_, *tetR*, pBL1 oriV*_Cg_*, dual-inducible *C. glutamicum*/*E. coli* expression shuttle vector	[69]
pRG_Duet2-*ddsA_Pd_*-*ubiA*	Kan^R^, pRG_Duet2 overexpressing *ddsA* from *P. denitrificans* (induced by IPTG) and *ubiA* from *E. coli* (induced by ATc)	[20]
pRG_Duet2-*ddsA_At_*-*ubiA*	Kan^R^, pRG_Duet2 overexpressing *ddsA* from *A. tumefaciens* (induced by IPTG) and *ubiA* from *E. coli* (induced by ATc)	This work
pRG_Duet2-*ddsA_Rs_*-*ubiA*	Kan^R^, pRG_Duet2 overexpressing *ddsA* from *R. sphaeroides* (induced by IPTG) and *ubiA* from *E. coli* (induced by ATc)	This work
pEC-XT99A	Tet^R^, P*_trc_*, *lacI^q^*, pGA1 oriV*_Cg_*, *C. glutamicum*/*E. coli* expression shuttle vector	[70]
pEC-XT99A-*ubiDIBX*	Tet^R^, pEC-XT99A overexpressing *ubiD*, *ubiI*, *ubiB*, and *ubiX* from *E. coli*	[20]
pEKEx3	Spec^R^, P*_tac_*, *lacI^q^*, pBL1 oriV*_Cg_*, *C. glutamicum*/*E. coli* expression shuttle vector	[71]
pEKEx3-*ubiGHEF*	Spec^R^, pEKEx3 overexpressing *ubiG*, *ubiH*, *ubiE*, and *ubiF* from *E. coli*	[20]
pK19*mobsacB*	Kan^R^, pK19 oriV*_Ec_*, *sacB*, *lacZα*, *E. coli*/*C. glutamicum* shuttle vector for construction of insertion and deletion mutants in *C. glutamicum*	[75]
pK19*mobsacB*-Δ*actA*:*ubiJK*	pK19*mobsacB* with a construct for deletion of *actA* (cg2840) and insertion of *ubiJ* and *ubiK* from *E. coli* under control of the native *actA* promoter	This work
pK19*mobsacB*-Δ*ispB*:P*_tuf_*-*ddsA_Pd_*	pK19*mobsacB* with a construct for deletion of *ispB* (cg0559) and insertion of *ddsA* from *P. denitrificans* under control of *C. glutamicum* promoter P*_tuf_*	This work

**Table 6:** Primers used in this study.

Primers	Sequence (5′ to 3′)
ddsA_At-fw	CCTGCAGGTCGACTCTAGAG**GAAAGGAGGCCCTTCAG**ATGGGCGTCGTCATACCGCTTG
ddsA_At-rv	GAGCTCGGTACCCGGGGATCTTAGTTGAGACGCTCGATGCAG
ddsA_Rs-fw	CCTGCAGGTCGACTCTAGAG**GAAAGGAGGCCCTTCAG**ATGGGATTGGACGAGGTTTC
ddsA_Rs-rv	GAGCTCGGTACCCGGGGATCTTAGGCGATGCGTTCGAC
actA-US-fw	GCATGCCTGCAGGTCGACTCTAGAGTCCCGTGCGTTGCATTTCCTG
actA-US-rv	CGGTTTCTAAACCAAGAAAAAACGGATCCCAGGTAATCGGACTTTTTCAAATTTTTCCC
actA-DS-fw	ATTTGAAAAAGTCCGATTACCTGGGATCCGTTTTTTCTTGGTTTAGAAACCG
actA-DS-rv	AATTCGAGCTCGGTACCCGGGGATCAGCCAATCGTCGTAAAGCG
ubiJ-fw	AATTTGAAAAAGTCCGATTACCTGG**CTCCCCCTTAGTAGAAAAGGAGGTTTTTCT**ATGCCTTTTAAACCTTTAGTGACG
ubiJ-rv	CTCAATTTTTTTCGGGTCAATCATCTGAAGGGCCTCCTTTCTCATTTAGCCTCCAGTTTTTCC
ubiK-fw	GGAAAAACTGGAGGCTAAATGA**GAAAGGAGGCCCTTCAG**ATGATTGACCCGAAAAAAATTGAG
ubiK-rv	TTTCTAAACCAAGAAAAAACGGATCTTACAGCGTTGGGGGGAGAG
actA-conf-fw	TTTCATCCGGCGCGAAGGTG
actA-conf-rv	GCTTCTGCGCAAAGCAAGCC
pSH1-ddsA-fw	CCTGCAGGTCGACTCTAGAG**GAAAGGAGGCCCTTCAG**ATGGGCATGAACGAAAACGT
pSH1-ddsA-rv	GAGCTCGGTACCCGGGGATCTTAGGACAGGCGCGAGACGA
ispB-US-fw	CCTGCAGGTCGACTCTAGAGTCATGAGATTTTGCCAAGCGG
ispB-US-rv	GGTTAAGTGGTGGATTACGGGGACTAGTTCATCGCTACCTTTGGTGATCG
ispB-DS-fw	CGATCACCAAAGGTAGCGATGAACTAGTCCCCGTAATCCACCACTTAACC
ispB-DS-rv	GAGCTCGGTACCCGGGGATCTATGAGAAGTCAGCACACGC
Ptuf-ddsA-fw	CTCGATCACCAAAGGTAGCGATGAATGGCCGTTACCCTGCGAATG
Ptuf-ddsA-rv	TTAAGTGGTGGATTACGGGGACTAGTTAGGACAGGCGCGAGACGAC
ispB-conf-fw	ATCACATGCTTCGCCTTGAC
ispB-conf-rv	TTTCTCGAAGGCAACACCTC

Ribosomal binding sites are in bold, binding regions of Gibson primers are underlined.

## Data Availability

The data presented in this study are available in article and supplementary material.

## Supplementary Materials

The following supporting information can be downloaded at: https://www.mdpi.com/article/10.3390/metabo12050428/s1, Figure S1: Mass spectra and SIM chromatograms for MK_8_(H_2_) and MK_9_(H_2_) in strains WT, UBI401, UBI405, UBI412, and UBI413; Figure S2: Mass spectra and SIM chromatograms for MK_10_(H_2_) and MK_11_(H_2_) in strains WT, UBI401, UBI405, UBI412, and UBI413; Figure S3: Mass spectra and SIM chromatograms for 8-11P-HB in strains WT, UBI401, UBI405, UBI412, and UBI413; Figure S4: Mass spectra and SIM chromatograms for 9-11P-Ph in strains WT, UBI401, UBI405, UBI412, and UBI413; Figure S5: Mass spectra and SIM chromatograms for MK_8–11_ in strains WT, UBI401, UBI405, UBI412, and UBI413; Figure S6: Mass spectra and SIM chromatograms for CoQ_8–11_ in strains WT, UBI401, UBI405, UBI412, and UBI413; Figure S7: Growth of *C. glutamicum* WT and the strains UBI4 and UBI5 in CGXII minimal medium with 40 g L^−1^ glucose in the BioLector microcultivation system; Figure S8: Overlay of SIM chromatograms from extracts of strains UBI4-Pd and UBI5-Pd for CoQ_8_, CoQ_11_, MK_10_(H_2_), and MK_10_(H_2_). Figure S9: Overlay of SIM chromatograms from extracts of strains UBI4-Pd and UBI4JK-Pd for CoQ_8_, CoQ_9_, MK_9_(H_2_), and MK_10_(H_2_). Figure S10: Overlay of SIM chromatograms from extracts of strains UBI6-Pd, UBI6-At, and UBI6-Rs for 10P-Ph.
