# Supplementary material for: Rational Engineering of Non-Ubiquinone Containing Corynebacterium glutamicum for Enhanced Coenzyme Q10 Production
Source: Metabolites. 2022 May 11;12(5):428. doi: 10.3390/metabo12050428 (PMC9145305; doi:10.3390/metabo12050428)
Supplement: Supplementary file 1 [file metabolites-12-00428-s001.zip › metabolites-1723845-supplementary.pdf]

Supplementary data

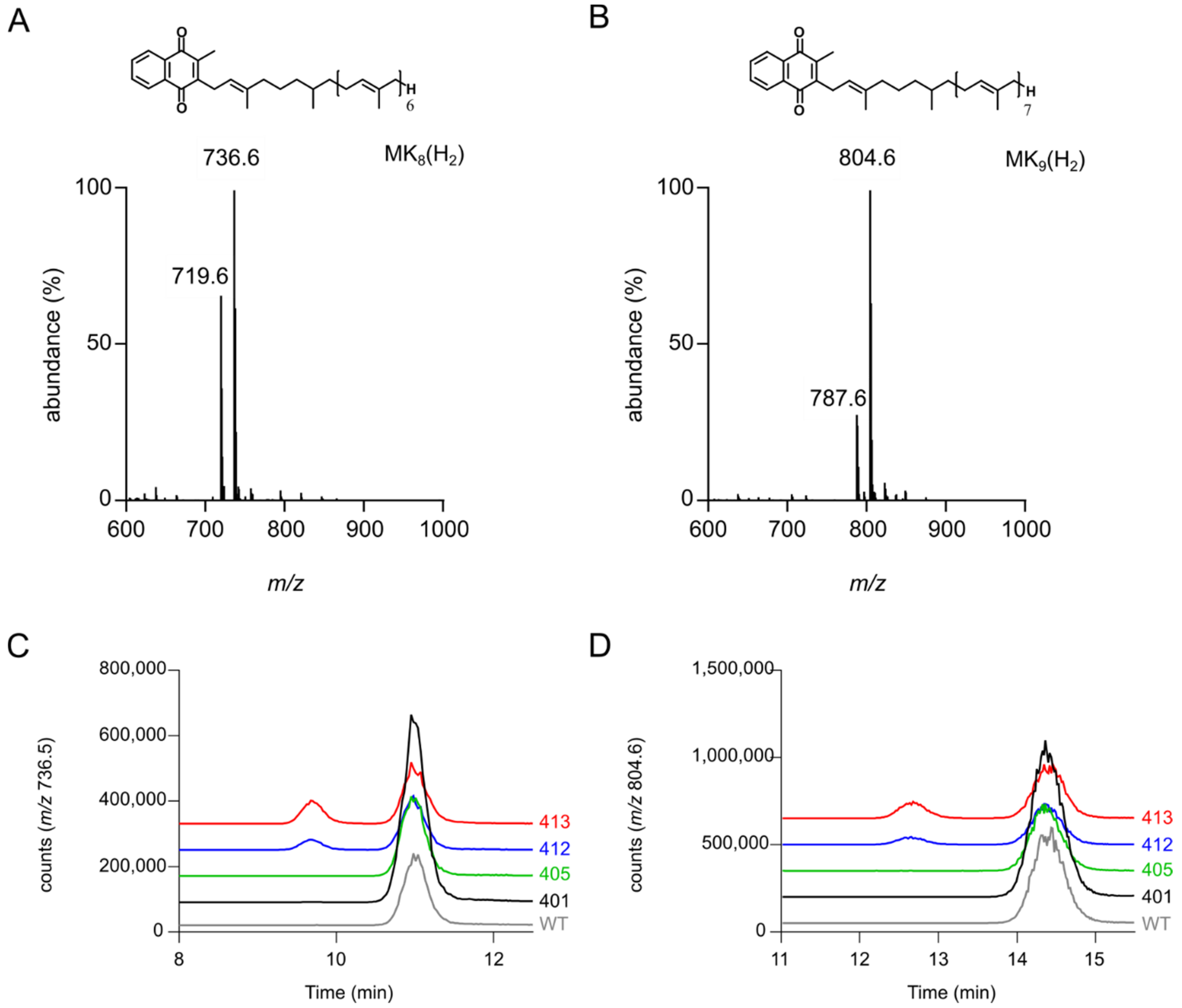

Figure S1. (**A**) Mass spectrum and structure of $MK_8(H_2)$ eluting at 11 min with m/z = 719.6 for the H^+ adduct and m/z = 736.6 for the NH_4^+ adduct. (**B**) Mass spectrum and structure of $MK_9(H_2)$ eluting at 14.4 min with m/z = 787.6 for the H^+ adduct and m/z = 804.6 for the NH_4^+ adduct. (**C–D**) Overlay of single ion monitoring (SIM) chromatograms for $MK_8(H_2)$ (NH_4^+ adduct m/z = 736.6, C) and $MK_9(H_2)$ (NH_4^+ adduct m/z = 804.6, D) in lipid extracts from the indicated strains (WT, UBI401, UBI405, UBI412, UBI413). Chromatograms are representative of three independent samples.

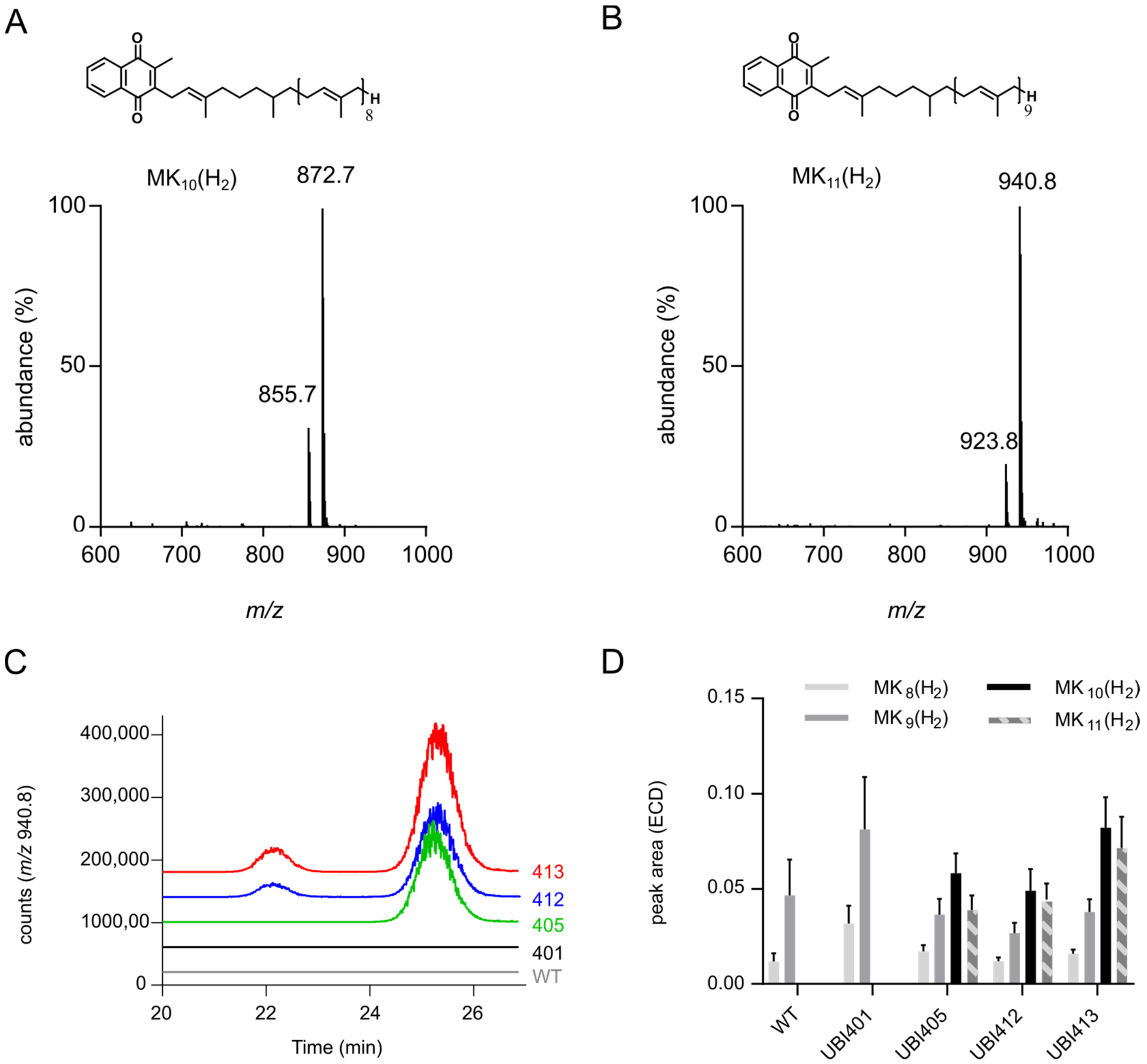

Figure S2. (**A**) Mass spectrum and structure of $MK_{10}(H_2)$ eluting at 19 min in sample UBI413 with m/z = 855.7 for the H^+ adduct and m/z = 872.7 for the NH_4^+ adduct. (**B**) Mass spectrum and structure of $MK_{11}(H_2)$ eluting at 25.3 min in sample UBI413 with m/z = 923.8 for the H^+ adduct and m/z = 940.8 for the NH_4^+ adduct. (**C**) Overlay of SIM chromatograms for $MK_{11}(H_2)$ (NH_4^+ adduct m/z = 940.8). Chromatograms are representative of three independent samples. (**D**) Quantification of peak area of $MK_{8\text{-}11}(H_2)$ in electrochemical detection (ECD) chromatograms of lipid extracts corresponding to 2 mg of cells, mean ± SD (n=3).

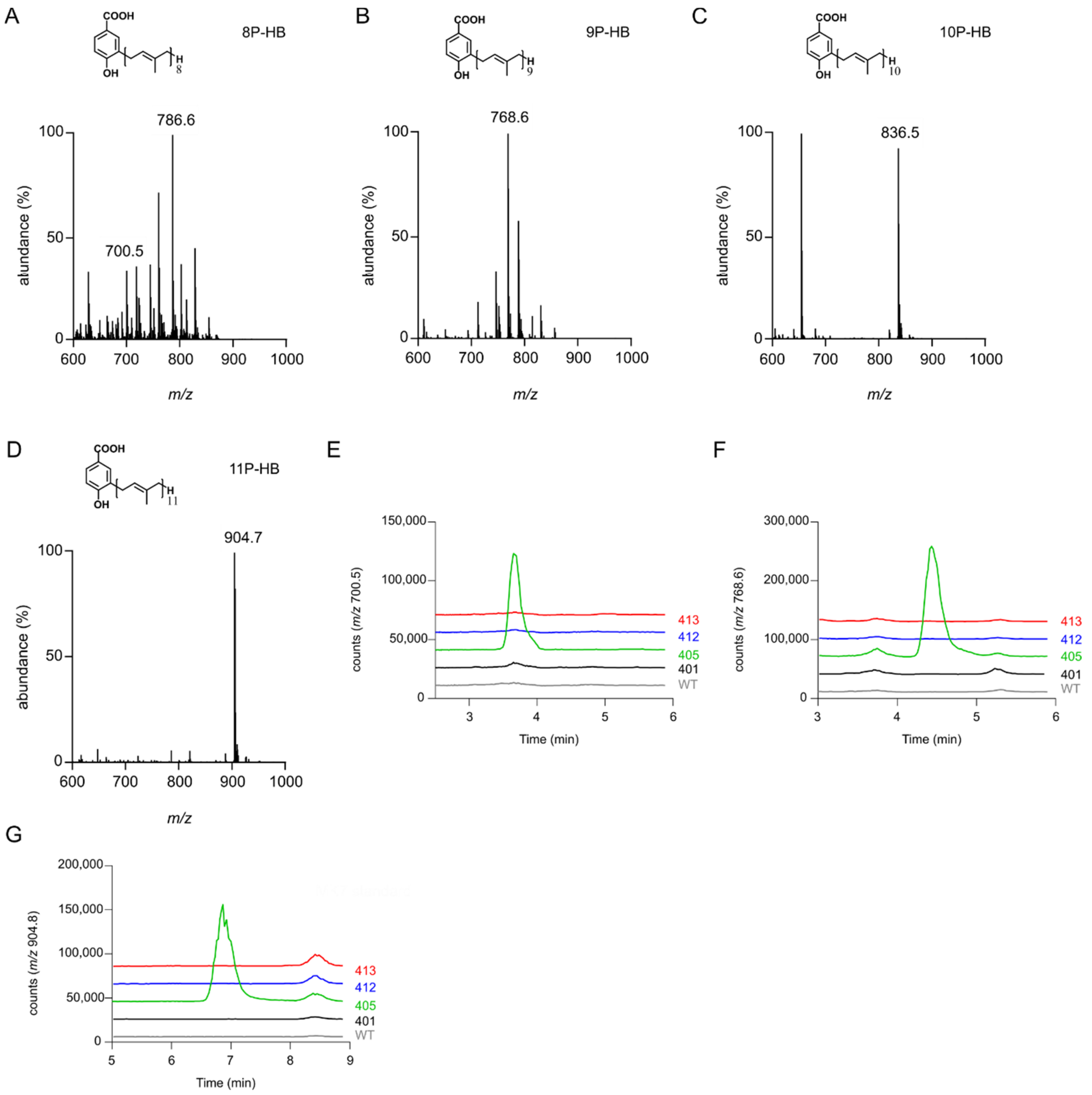

Figure S3. (**A**) Mass spectrum and structure of 8P-HB eluting at 3.8 min in sample UBI405 with m/z = 700.5 for the NH_4^+ adduct. The complexity of the mass spectrum reveals the presence of other co-eluting molecules. (**B**) Mass spectrum and structure of 9P-HB eluting at 4.5 min in sample UBI405 with m/z = 768.6 for the NH_4^+ adduct. (**C**) Mass spectrum and structure of 10P-HB eluting at 5.5 min in sample UBI405 with m/z = 836.6 for the NH_4^+ adduct. (**D**) Mass spectrum and structure of 11P-HB eluting at 6.9 min in sample UBI405 with m/z = 904.7 for the NH_4^+ adduct. (**E–G**) Overlay of SIM chromatograms for 8P-HB (NH_4^+ adduct m/z = 700.5, E), 9P-HB (NH_4^+ adduct m/z = 768.6, F), and 11P-HB (NH_4^+ adduct m/z = 904.8, G). Chromatograms are representative of three independent samples.

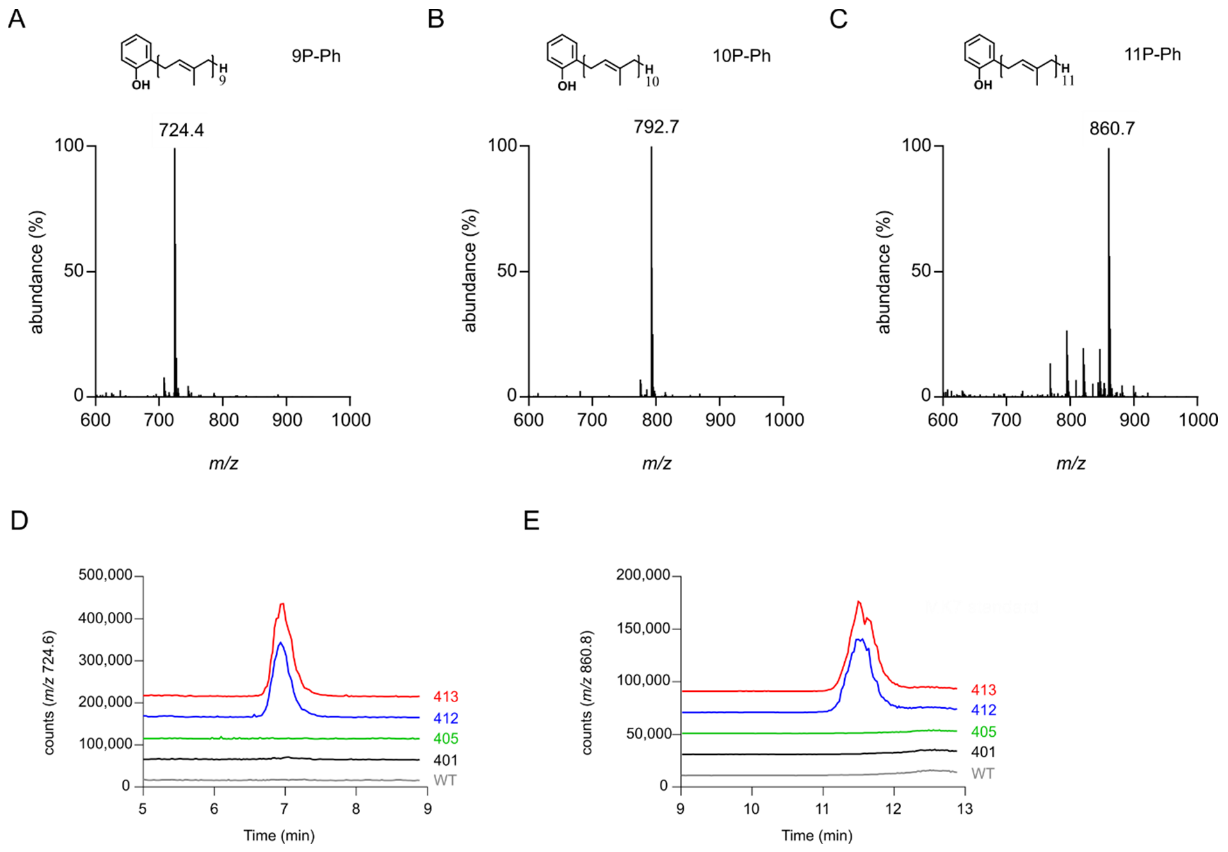

Figure S4. (**A**) Mass spectrum and structure of 9P-Ph eluting at 6.9 min in sample UBI412 with m/z = 724.6 (NH_4^+ adduct). (**B**) Mass spectrum and structure of 10P-Ph eluting at 8.8 min in sample UBI412 with m/z = 792.7 (NH_4^+ adduct). (**C**) Mass spectrum and structure of 11P-Ph eluting at 11.5 min in sample UBI412 with m/z = 860.7 (NH_4^+ adduct). (**D–E**) Overlay of SIM chromatograms for 9P-Ph (NH_4^+ adduct m/z = 724.6, D) and 11P-Ph (NH_4^+ adduct m/z = 860.8, E). Chromatograms are representative of three independent samples.

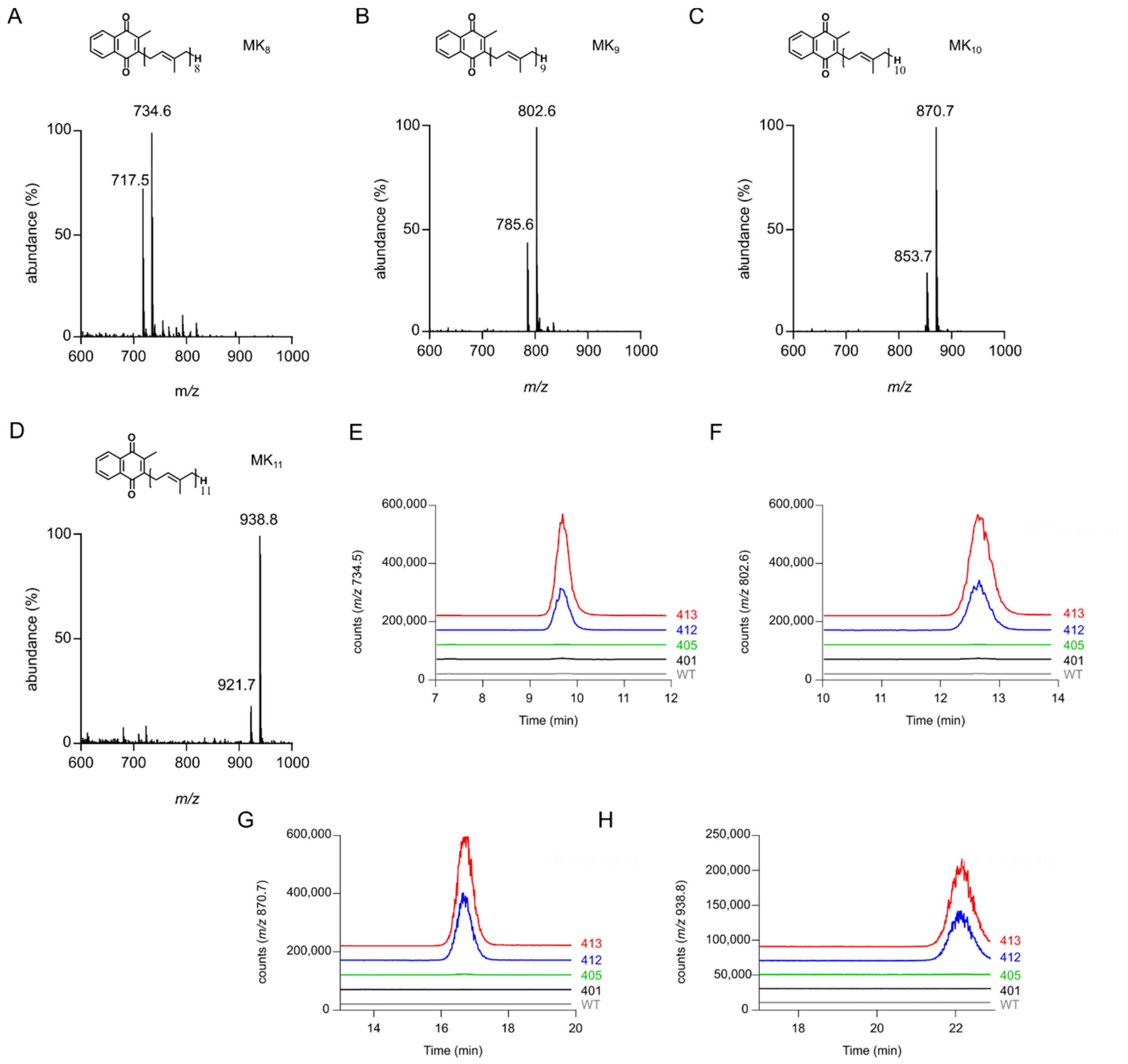

Figure S5. (**A**) Mass spectrum and structure of MK_8 eluting at 9.6 min in sample UBI413 with m/z = 717.6 for the H^+ adduct and m/z = 734.6 for the NH_4^+ adduct. (**B**) Mass spectrum and structure of MK_9 eluting at 12.7 min in sample UBI413 with m/z = 785.6 for the H^+ adduct and m/z = 802.6 for the NH_4^+ adduct. (**C**) Mass spectrum and structure of MK_{10} eluting at 16.5 min in sample UBI413 with m/z = 853.7 for the H^+ adduct and m/z = 870.7 for the NH_4^+ adduct. (**D**) Mass spectrum and structure of MK_{11} eluting at 22.2 min in sample UBI413 with m/z = 921.8 for the H^+ adduct and m/z = 938.8 for the NH_4^+ adduct. (**E–H**) Overlay of SIM chromatograms for MK_8 (NH_4^+ adduct m/z = 734.5, E), MK_9 (NH_4^+ adduct m/z = 802.6, F), MK_{10} (NH_4^+ adduct m/z = 870.7, G), and MK_{11} (NH_4^+ adduct m/z = 938.8, H). Chromatograms are representative of three independent samples.

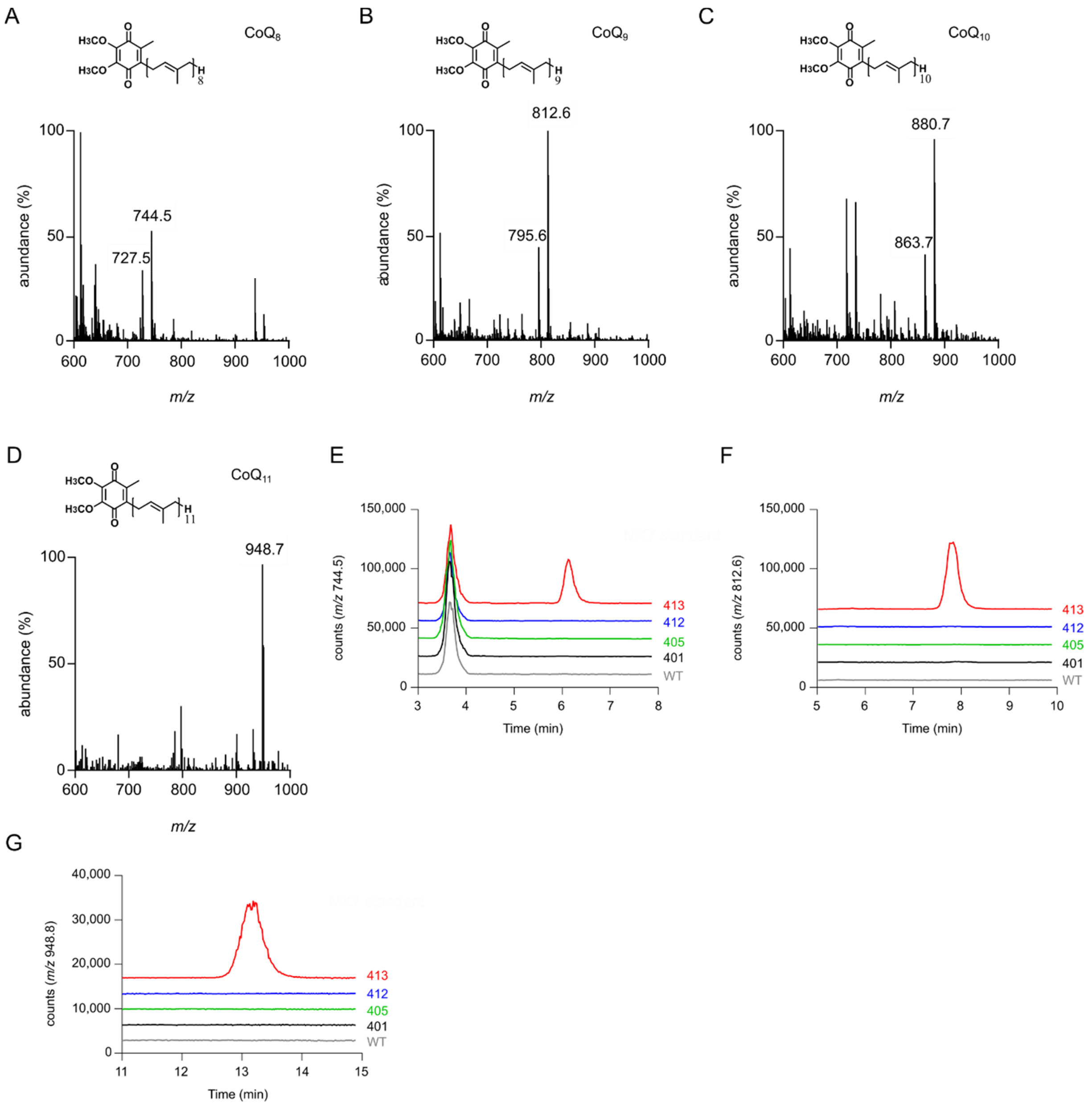

Figure S6. (**A**) Mass spectrum and structure of CoQ_8 eluting at 6.2 min in sample UBI413 with m/z = 727.5 for the H^+ adduct and m/z = 744.5 for the NH_4^+ adduct. (**B**) Mass spectrum and structure of CoQ_9 eluting at 7.9 min in sample UBI413 with m/z = 795.6 for the H^+ adduct and m/z = 812.6 for the NH_4^+ adduct. (**C**) Mass spectrum and structure of CoQ_{10} eluting at 10 min in sample UBI413 with m/z = 863.7 for the H^+ adduct and m/z = 880.7 for the NH_4^+ adduct. (**D**) Mass spectrum and structure of CoQ_{11} eluting at 13.2 min in sample UBI413 with m/z = 948.8 for the NH_4^+ adduct. (**E–G**) Overlay of SIM chromatograms for CoQ_8 (NH_4^+ adduct m/z = 744.5, E), CoQ_9 (NH_4^+ adduct m/z = 812.6, F), and CoQ_{11} (NH_4^+ adduct m/z = 948.8, G). Chromatograms are representative of three independent samples.

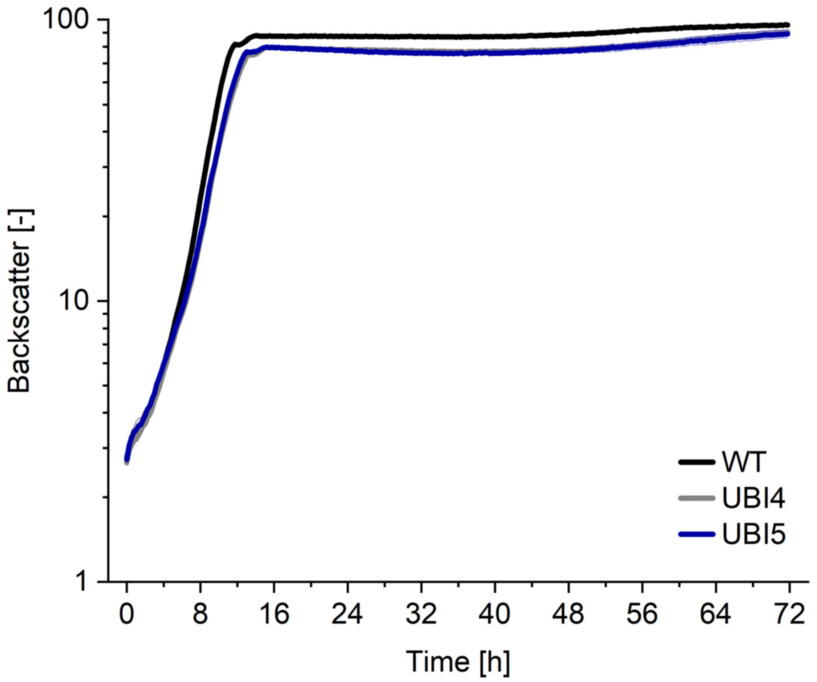

Figure S7. Growth of *C. glutamicum* WT and the strains UBI4 and UBI5 in CGXII minimal medium with 40 g L^{-1} glucose in the BioLector microcultivation system. Values and error bands represent means and standard deviations of 3 independent cultivations.

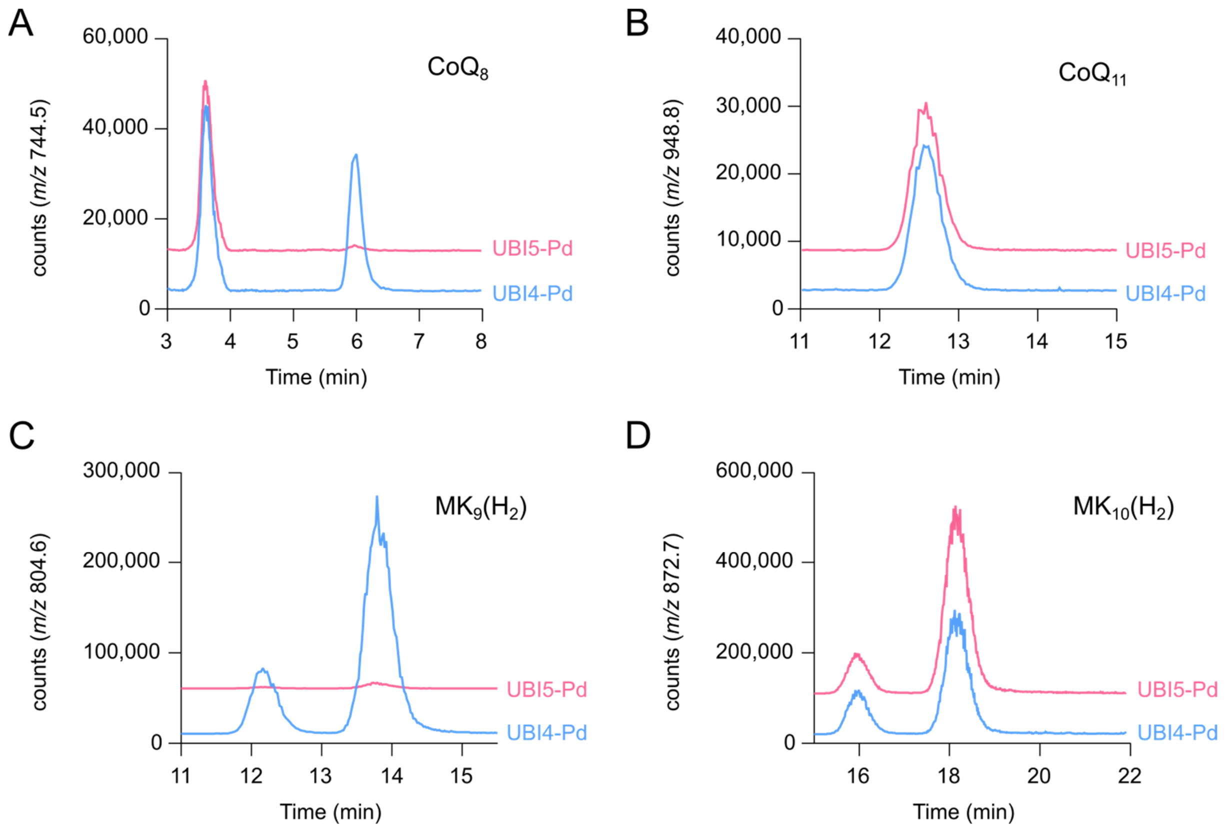

Figure S8. (**A–D**) Overlay of SIM chromatograms from extracts of strains UBI4-Pd and UBI5-Pd for CoQ_8 (NH_4^+ adduct m/z = 744.5, A), CoQ_{11} (NH_4^+ adduct m/z = 948.8, B), $MK_9(H_2)$ (NH_4^+ adduct m/z = 804.6, C), and $MK_{10}(H_2)$ (NH_4^+ adduct m/z = 872.7, D). Chromatograms are representative of three independent samples.

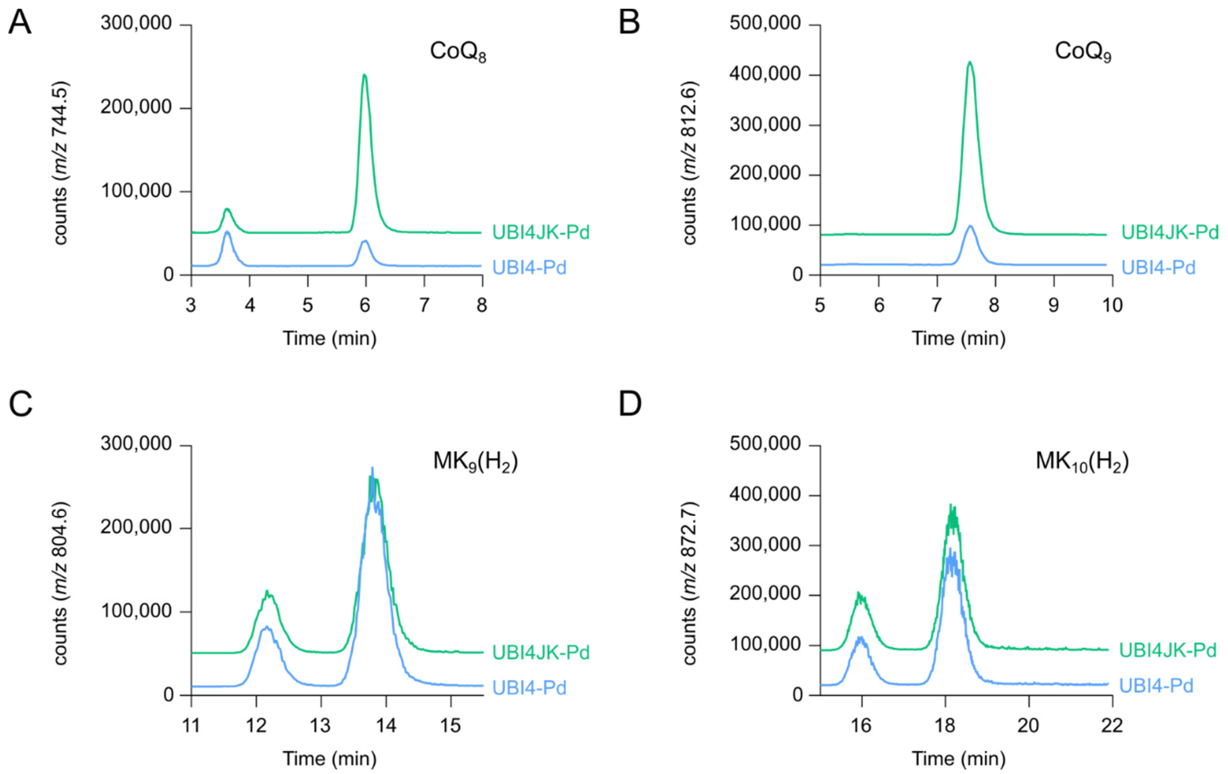

Figure S9. (**A–D**) Overlay of SIM chromatograms from extracts of strains UBI4-Pd and UBI4JK-Pd for CoQ_8 (NH_4^+ adduct m/z = 744.5, A), CoQ_9 (NH_4^+ adduct m/z = 812.6, B), $MK_9(H_2)$ (NH_4^+ adduct m/z = 804.6, C), and $MK_{10}(H_2)$ (NH_4^+ adduct m/z = 872.7, D). Chromatograms are representative of three independent samples.

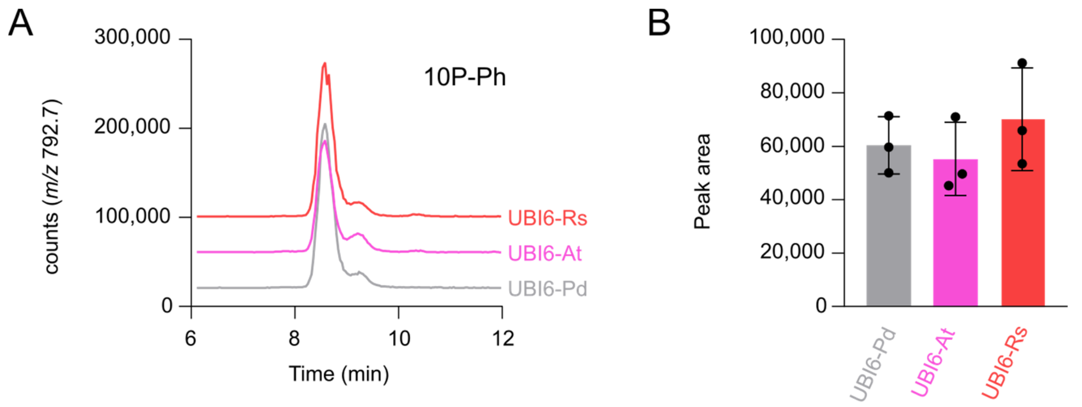

Figure S10. (**A**) Overlay of SIM chromatograms from extracts of strains UBI6-Pd, UBI6-At, and UBI6-Rs for 10P-Ph (NH_4^+ adduct m/z = 792.7). Chromatograms are representative of three independent samples. (**B**) Quantification of 10P-Ph (MS peak area) in three independent samples UBI6-Pd, UBI6-At, and UBI6-Rs, mean ± SD (n = 3).
